# Disruption of the inositol phosphorylceramide synthase gene affects *Trypanosoma cruzi* differentiation and infection capacity

**DOI:** 10.1371/journal.pntd.0011646

**Published:** 2023-09-20

**Authors:** Nailma S A Dos Santos, Carlos F. Estevez-Castro, Juan P. Macedo, Daniela F. Chame, Thiago Castro-Gomes, Mariana Santos-Cardoso, Gabriela A. Burle-Caldas, Courtney N. Covington, Patrick G. Steel, Terry K. Smith, Paul W. Denny, Santuza M. R. Teixeira

**Affiliations:** 1 Departamento de Bioquímica e Imunologia, Universidade Federal de Minas Gerais, Belo Horizonte, Brazil; 2 Departamento de Parasitologia, Universidade Federal de Minas, Belo Horizonte, Brazil; 3 Department of Chemistry and Centre for Global Infectious Disease, Durham University, Durham, United Kingdom; 4 BSRC School of Biology, Biomolecular Science Building, St Andrews, United Kingdom; 5 Department of Biosciences and Centre for Global Infectious Diseases, Durham University, Durham, United Kingdom; Solena Ag, UNITED STATES MINOR OUTLYING ISLANDS

## Abstract

Sphingolipids (SLs) are essential components of all eukaryotic cellular membranes. In fungi, plants and many protozoa, the primary SL is inositol-phosphorylceramide (IPC). *Trypanosoma cruzi* is a protozoan parasite that causes Chagas disease (CD), a chronic illness for which no vaccines or effective treatments are available. IPC synthase (IPCS) has been considered an ideal target enzyme for drug development because phosphoinositol-containing SL is absent in mammalian cells and the enzyme activity has been described in all parasite forms of *T*. *cruzi*. Furthermore, IPCS is an integral membrane protein conserved amongst other kinetoplastids, including *Leishmania major*, for which specific inhibitors have been identified. Using a CRISPR-Cas9 protocol, we generated *T*. *cruzi* knockout (KO) mutants in which both alleles of the IPCS gene were disrupted. We demonstrated that the lack of IPCS activity does not affect epimastigote proliferation or its susceptibility to compounds that have been identified as inhibitors of the *L*. *major* IPCS. However, disruption of the *T*. *cruzi* IPCS gene negatively affected epimastigote differentiation into metacyclic trypomastigotes as well as proliferation of intracellular amastigotes and differentiation of amastigotes into tissue culture-derived trypomastigotes. In accordance with previous studies suggesting that IPC is a membrane component essential for parasite survival in the mammalian host, we showed that *T*. *cruzi* IPCS null mutants are unable to establish an infection *in vivo*, even in immune deficient mice.

## Author summary

*Trypanosoma cruzi* is a vector-borne parasite that causes Chagas disease (CD), an illness that affects 6–8 million people worldwide and results in approximately 50,000 deaths per year. Transmitted by more than 100 species of triatomine insects, *T*. *cruzi* has a complex life cycle that includes parasite forms that proliferate in the gut of the insect vector (epimastigotes) and in the cytoplasm of infected mammalian cells (amastigotes). To be transmitted to mammals, epimastigotes must differentiate into metacyclic trypomastigotes, and to infect new mammalian cells, amastigotes must differentiate into bloodstream trypomastigotes. Sphingolipids (SLs) are essential components of all eukaryotic cellular membranes and are present in all forms of *T*. *cruzi* as part of various cell surface molecules involved in host-parasite interactions. Different from mammals, in which sphingomyelin is the main SL, *T*. *cruzi* synthesizes inositol phosphorylceramide (IPC) and, because of that, enzymes of the SL biosynthetic pathway have been considered potential targets for the development of new treatments for CD. By disrupting the gene encoding the *T*. *cruzi* IPC synthase, we showed that parasite differentiation from epimastigotes into metacyclic trypomastigotes and from amastigotes to bloodstream forms are affected and, as a consequence, these knockout cell lines are unable to establish an infection in animals.

## Introduction

*Trypanosoma cruzi* is a vector-borne flagellated parasite that belongs to the order Kinetoplastida [1]. It is the causative agent of Chagas disease, an anthropozoonosis that affects about 6–8 million people worldwide and causes approximately 50,000 deaths per year [2]. Although Chagas disease was once entirely confined to Central and South America’s countries in which the parasite is endemic, in our days, it has spread to non-endemic regions such as North America, Europe, and Western Pacific regions due to migration of infected individuals and current estimations indicate that approximately 75 million people worldwide are at risk of contracting Chagas disease [3,4].

*T*. *cruzi* is primarily transmitted to humans by triatomine insect vectors, also known as “kissing bugs” but non-vectorial mechanisms of infection can also occur through organ transplantations, blood transfusions, oral and congenital transmission routes [4]. The parasite has a complex life cycle with four morphologically and physiologically different forms that alternate between the blood-sucking triatomine vectors and mammalian hosts. Inside the insect midgut, epimastigote forms proliferate until reaching the hindgut, where they differentiate into non-dividing infectious metacyclic trypomastigotes. During the insect blood meal, metacyclic trypomastigotes are transmitted to mammals by the contaminated feces that pass through the skin lesions caused by the vector’s bite. After infection, metacyclic trypomastigotes penetrate different types of host cells and transform into replicative amastigotes. In the cytoplasm of host cells, the amastigotes multiply by binary fission and differentiate into non-replicative trypomastigotes, which are released to the bloodstream upon host cell lysis. Infective bloodstream trypomastigotes can spread and invade a range of nucleated cells or can be taken up by another insect, together with the infected blood to restart a new cycle [4,5].

Despite more than a hundred years of research, a vaccine to prevent Chagas disease is not available, and treatment relies on two drugs, benznidazole (BZ) and nifurtimox (NF), both with substantial limitations [5]. Treatment is often discontinued due to adverse side effects and inefficacy against the genetic background of the infecting strain [6]. The lack of effective therapy or vaccine demands an intensive search for alternative anti-trypanosomal therapies with alternative molecular targets that may allow developing effective treatment and control.

Sphingolipids (SL) represent one of the major classes of eukaryotic lipids and, because they have key cellular functions, selective inhibitors of SL biosynthesis have been tested as target candidates for the development of drugs against pathogenic fungi and protozoan parasites, as well as its use as a herbicide [7,8]. Initially considered molecules of structural importance only, over the last two decades, numerous studies have shown new roles for SLs related to cell growth regulation, polarized cellular trafficking, signal transduction, differentiation and apoptosis [9]. Structurally, SLs can be distinguished from other lipids by the presence of a sphingoid base backbone, sphingosine, that can be acylated to form N-acyl-sphingosines, commonly named ceramides. The combination of ceramide and phosphorylcholine forms sphingomyelin (SM), the predominant complex SL in mammals, whilst in yeast, plants and protozoa the most common SL is inositol phosphorylceramide (IPC), a combination of inositol phosphate and ceramide [10]. Such a structure confers an amphipathic character to SLs which, together with their higher than average hydrophobic lipid moiety, results in their tendency to aggregate into microdomains within membranes.

Mammalian sphingolipid metabolism has been extensively studied and all the key enzymes have been identified. Briefly, *de novo* biosynthesis takes place in the endoplasmic reticulum (ER) and Golgi apparatus [11] and can be simplified into three crucial steps: i) the rate-limiting step, that involves the condensation of acyl-CoA and L-serine in the ER via serine palmitoyltransferase (SPT) to produce 3-ketodihydrosphingosine; ii) acylation of 3-dihydrosphingosine to dihydroceramide by the action of the ceramide synthase, which is later oxidized to form ceramide; iii) formation of complex SLs from ceramide via the activity of SL synthases, reactions that takes place in the Golgi apparatus. Different studies have shown evidence for the presence of these three reactions in the trypanosomes [12–15], but the product of the third reaction is significantly different. Whilst in mammals ceramide and phosphatidylcholine are the substrates for sphingomyelin synthase (SMS) to produce SM, in fungi, plants and protozoa ceramide and phosphatidylinositol are utilized by inositol phosphorylceramide synthase (IPCS) to form IPC [10]. As phosphoinositol-containing SL is absent in mammalian cells, the IPC synthase (IPCS) enzyme has been considered a potential molecular target for drug development in both fungi and protozoa. In yeast, *IPCS* (AUR1) is an essential gene and inhibitors of the encoding enzyme have potent fungicidal activity [16]. In *T*. *cruzi*, IPC is part of glycoinositolphospholipids (GIPLs) as well as glycosylphosphatidyl inositol (GPI) anchors of various glycoproteins such as *trans*-sialidases and mucins present on the cell surface of all forms of the parasite [17–19]. Changes in GPI anchors have been associated with differentiation from the insect form epimastigotes to infective metacyclic trypomastigote forms: distinct from the phosphatidylinositol moiety of mucins present on the surface of epimastigotes, the inositol lipid moiety of mucins in metacyclic trypomastigotes contains mostly IPC [20].

Research towards drug development using protozoan IPCS as target has been benefitted by studies with the *Leishmania major* IPCS enzyme, which is able to complement for the absence of the *Saccharomyces cerevisiae* orthologue AUR1 [15]. Using a yeast-based high throughput screening assay, antileishmanial benzazepanes were identified as potent and specific inhibitors of the *Leishmania* IPCS [15,21]. In contrast to the *L*. *major* enzyme, attempts to functionally validate the *T*. *cruzi* IPCS (TcIPCS) using yeast complementation assays were not successful [22]. To gain further insights into the role of the TcIPCS and to investigate its potential as a drug target in *T*. *cruzi*, we took advantage of the CRISPR-Cas technology to disrupt the gene encoding TcIPCS and investigate the consequences of the absence of IPC for parasite virulence. We showed that, whilst the absence of TcIPCS activity does not affect epimastigote proliferation or susceptibility to compounds known to target the *L*. *major* IPCS, parasite differentiation and the capacity to complete the intracellular cycle is severely impaired. In addition to showing that *TcIPCS* disruption inhibits amastigote proliferation and differentiation into trypomastigotes *in vitro*, we demonstrated that *TcIPCS* null mutants were unable to cause infection *in vivo*, even in highly susceptible animals. Together, our data support previous studies suggesting that IPC is a membrane component essential for parasite survival in the mammalian host and, therefore, IPCS is a key enzyme for *T*. *cruzi* virulence.

## Methods

### Structural predictions

Nucleotide and amino acid sequences with the following accession numbers were retrieved from TriTrypDB (www.tritrydb.org): *L*. *major IPCS*, LmjF.35.4990; *T*. *brucei SLS1-4*, Tb927.9.9410, Tb927.9.9400, Tb927.9.9390 and Tb927.9.9380; *TcIPCS*, TcCLB.506885.124 (esmeraldo-like allele), TcCLB.510729.290 (non-esmeraldo allele). These sequences have been previously identified as kinetoplastid sphingolipid synthases [14]. The three dimensional (3D) structures were modeled using trRosetta (Yang-server) [23] and visualized using Pymol v2.4.1 (Schrödinger, LLC). Topology and active site prediction were based on [24] and adjusted by the modeled structure. The amino acid sequence alignment was made using TM aligner [25] and plotted using the ESPript server 3.0 available at http://espript.ibcp.fr/.

### Parasite cultures

*Trypanosoma cruzi* epimastigotes of the CL Brener strain were maintained in logarithmic growth at 28°C in liver infusion tryptose (LIT) medium, supplemented with 10% heat-inactivated fetal bovine serum (GIBCO), penicillin (100 U/mL) and streptomycin (100 U/mL) (GIBCO) as previously described [26]. Metacyclic trypomastigotes, obtained after metacyclogenesis of epimastigotes maintained in LIT medium for 15 days, were used to infect Rhesus Monkey Kidney Epithelial Cells (LLC-MK2) grown in Dulbecco’s Modified Eagle Medium (DMEM), supplemented with 5% inactivated fetal bovine serum (FBS) at 37°C and 5% CO2 [27]. After 4 hours of infection, the cells were washed with PBS (132 mM NaCl; 3 mM KCl; 8 mM Na2HPO4; 1,5 mM KH2PO4; pH 7,2) and maintained in fresh DMEM medium supplemented with 5% non-inactivated horse serum (GIBCO) to lyse epimastigote forms. Four days post-infection (dpi), LLC-MK2 cultures were washed and maintained in DMEM medium supplemented with 2% FBS at 34°C and 5% CO2. Tissue-culture derived trypomastigotes (TCT) were collected from the infected monolayers, centrifuged for 10 min at 600 x g, and then allowed to swim up away from the pellet for 6–12 hours in a 34°C incubator. Purified, highly motile trypomastigotes collected from the supernatant were used for *in vitro* infection assays.

### Disruption of the *T*. *cruzi IPCS* gene

The design of single guide RNA and *TcIPCS* gene knockout (KO) using CRISPR-Cas9 protocols were followed as previously described [28]. Briefly, wild type epimastigotes were transfected with *Staphylococcus aureus* recombinant Cas9 (rSaCas9), purified from *E*. *coli*, and complexed with two single guide RNAs (sgRNAs) that were transcribed *in vitro*. In addition, a PCR fragment containing a *neomycin phosphotransferase* (*NPT*) gene flanked by the 5’ and 3’ *TcIPCS* regions, which can be used for homology-directed repair (HDR) of the double-strand break was also transfected (see S1 Table for primers sequences). The sgRNAs were designed using the Eukaryotic Pathogen CRISPR guide RNA/DNA design tool (EuPaGDT), available at http://grna.ctegd.uga.edu/, and contains sequences homologous to the 5’ and 3’ ends of both alleles of the *TcIPCS* coding region. They were positioned at ~50 bp after the translation initiation codon and ~20 bp before the translation stop codon. Therefore, the action of both sgRNA guided the deletion of almost the entire *TcIPCS* coding sequence. For the construction of the plasmid containing the DNA donor for HDR, two sets of primers were designed (S1 Table) for the amplification of approximately 370 bp upstream and downstream from the *TcIPCS* coding region. For the upstream sequence, the forward and reverse primers contain KpnI and SpeI sites, respectively, and for the downstream sequence, the primers contain EcoRV and XhoI sites. These amplified regions were sequentially cloned into the restriction sites KpnI/SpeI and EcoRV/XhoI of the plasmid TopoHX1-Neo-GAPDH [29]. The resulting plasmid was used as template for PCR amplification of a 2561 bp fragment corresponding to the HDR DNA donor. Transfection conditions were as previously described [28] using the Amaxa Nucleofector (Lonza) program U-033. After two pulses, parasites were transferred to a flask with LIT medium and 200 mM of G418 (GIBCO) and cloned cell lines were generated on 96 well plates using the limiting dilution method. Two plasmid constructs were prepared for the generation of *TcIPCS* KO cell lines: TOPOHX1-NEO-GAPDH and TOPO-5’_UTR_IPCS-HX1-NEO-GAPDH-3’_UTR_IPCS. As indicated in the next section, two plasmids, pROCK-HX1-HYGROMYCIN-GAPDH and pROCK-HYGROMYCIN-IPCS-HA were used for the generation of *TcIPCS* addback cell lines.

### Generation of HA-tagged parasites and protein localization

The pROCK vector containing the full-length coding sequence of the *TcIPCS* gene with a 3’ HA tag at its C-terminal region was generated by ligating a PCR product containing this sequence into the XbaI and XhoI sites of the pROCK-hygromycin vector [30]. After NotI digestion, the linearized plasmid was transfected into epimastigotes cultures using previously described protocols. Hygromycin resistant epimastigotes were selected and used in immunofluorescence (IF) analyses with an anti-HA antibody. For IF assays, epimastigotes were harvested, centrifuged and washed in Hank’s balanced salt solution plus HEPES (HBSS/HEPES) and fixed with 4% paraformaldehyde. Fixed parasites were deposited on circular microscopy coverslips pre-treated with 0.1% poly-L-lysine solution (Sigma-Aldrich) in order to immobilize the epimastigotes on the coverslip and before starting the labelling process. The attached parasites were quenched with 50 mM ammonium chloride for 30 min at room temperature. After quenching, parasites were permeabilized with 0.1% TRITON X-100 (Sigma-Aldrich) diluted in phosphate-buffered saline (PBS) and incubated with blocking solution (PBS containing 1% bovine serum albumin, 0.1% saponin and 5% horse serum) for 60 min. After blocking, coverslips were washed twice with PBS and incubated for 60 min with 1:50 mouse anti-HA IgG, followed by washing and incubation with 1:250 secondary anti-IgG antibody conjugated with Alexa Fluor 488 (Life Technologies) for another 60 min. After a final washing with PBS, parasites were incubated with DAPI (1:1000) (Life Technologies) to stain cell nuclei and kinetoplasts. All antibodies and probes were diluted in blocking solution and microscopy slides were prepared using ProLong Gold Antifade Mounting Reagent (Life Technologies). For experiments using NBD C_6_-ceramide probe (Invitrogen), to stain the Golgi apparatus, the parasites were incubated with 5 μM of the probe at 4°C for 30 min. In the next step, the probe was removed and coverslips were washed and incubated for 90 min with HBSS/HEPES containing 10% fetal bovine serum, following by 30 min quenching with 50 mM ammonium chloride. Depending on the experiment, parasites could be labelled with both NBD C_6_-ceramide probe and with anti-HA/Alexa 488 IgG antibodies for immunofluorescence labelling. In this case, NBD C_6_-ceramide labelling was always performed first. Fluorescence microscopy analysis of the labelled parasites were performed using BX60 Upright compound fluorescence microscope (Olympus) or Axio Imager 2 ApoTome microscope (ZEISS). Images were acquired using the software Q-Capture (BX60 microscope) or ZEN—Blue Edition (ZEISS Axio Imager 2 ApoTome microscope). Images were prepared using ZEN Blue Edition software (ZEISS) and Image-J (NIH).

### RNA and DNA analyses

*T*. *cruzi* genomic DNA (CL Brener) was used as template for PCR amplifications, was purified from 10^8^ log-phase epimastigotes using the Illustra blood GenomicPrep Mini Spin Kit (GE Healthcare) following the manufacturer’s recommendations. PCR reactions used GoTaq DNA polymerase (Promega). The PCR reactions were incubated in the ProFlex PCR System (Applied Biosystems) with the following program: i) initial denaturation at 95°C for 1 min; ii) 30 cycles of denaturation at 95°C for 30 s, primer annealing at 50–64°C for 30 s, and extension time (1 min/Kb) either at 72°C for GoTaq or 68°C for TaKaRa LA Taq; and iii) final extension at 72°C for 10 min. The amplified products were analyzed by 1% agarose gel electrophoresis.

*TcIPCS* gene KO was confirmed by PCR using DNA purified from cloned cell lines and primers that anneal in both alleles, upstream and downstream of the inserted *Neo* resistance cassette. Primers that anneal in the *NPT* gene were also used (S1 Table). For quantitative RT-PCR analyses to determine *TcIPCS* mRNA levels, primers 16 and 17 (S1 Table) were used. Total RNA was isolated from 2 x 10^8^ log-phase epimastigotes using the TRIzol reagent protocol (Invitrogen) as recommended by the manufacturer. RNA was also isolated from tissue culture derived trypomastigotes collected from the supernatant of infected Vero cells as well as from extracellular amastigotes. Extracellular amastigotes were obtained by differentiating tissue culture derived trypomastigotes for 18–24 h in LIT medium at pH 5.8 supplemented with 5% FBS, according to previously published protocols [31]. Samples were treated with DNase I (Thermo Scientific) following manufacturer instructions and the RNA was quantified using the Qubit RNA BR assay kit in a Qubit 4 Fluorometer (Invitrogen). A SuperScript IV kit (Invitrogen) was used to synthesize the first strand cDNA, following the manufacturer recommendations with few modifications. The resulting cDNA solutions were diluted 1:10 in DNase free water and used as a template for the quantitative PCR reactions performed using SSO SYBR Green Supermix (Bio-Rad) with primers specific for the *TcIPCS* and the control genes *GAPDH* and *TcL9* [32] (S1 Table). Reactions were pipetted into a 96-well plate and analyzed on the Applied Biosystems 7900HT Fast Real-Time PCR System (Life Technologies). Relative *TcIPCS* mRNA levels were normalized against the Ct values of the gene encoding for constitutive ribosomal protein TcL9 or GAPDH, following the 2-∆∆ct method [33].

## Growth curve and *in vitro* infections

Log-phase epimastigotes were diluted in fetal bovine serum-supplemented LIT medium to an initial density of 2.5 x 10^6^ parasites/mL. Parasite density was determined by manual cell counting using a hemocytometer chamber. *In vitro* metacyclogenesis analyses were performed in 5 mL cultures in LIT medium incubated at 28°C for 9 days to allow differentiation by nutritional stress [26]. Subsequently, the cultures were homogenized, and 10 μL samples were smeared in poly-L-lysine coated glass slides, fixed with methanol for 5 min, followed by Giemsa staining. Samples were mounted with a drop of Entellan (Merck) and a glass coverslip and analyzed by light microscopy. The different stages of parasite differentiation were determined according to a previously described model [34]. At least 200 parasites were analyzed in each culture to determine the percentages of metacyclic trypomastigotes.

For *in vitro* infections, Vero cells were plated in 24-well plates and infected with purified TCT at a multiplicity of infection (MOI) of 10:1. Cell infection assays were performed in a plated monolayers at a density of 2.5 x 10^4^ cells/well during 4 h of infection, washed three times with PBS 1X, and incubated with DMEM 2% SBF at 37°C for 24 h. Intracellular amastigote proliferation was evaluated as above, with two additional incubation times (48 and 72 h). After incubation, infected monolayers were fixed and stained with a hematoxylin-eosin panoptic stain kit (RenyLab) and mounted on microscope slides with Entellan (Merk). The percentage of infected cells or the number of amastigotes per cell was quantified by direct counting in a hemocytometer chamber of at least 20 different fields of vision at the 100X objective that accounted for a minimum of 800 cells. To evaluate amastigote differentiation into TCTs and its release to the culture supernatant, 24-well plates containing 5x10^4^ cells/well were prepared and infected for 24 h. Afterward, infected monolayers were washed three times with PBS 1X and incubated with 2% FBS-DMEM at 37°C. Supernatant TCTs or extracellular amastigotes were determined over 12 days by direct counting in a hemocytometer chamber.

### *In vivo* infections

IFN-gamma-deficient (IFN-γ KO) mice [35], in C57BL/6 background (females, 8–12-week-old), were bred under specific pathogen free conditions (Fiocruz, Belo Horizonte, MG, Brazil). During the experiments, animals were kept in a conventional animal facility at controlled temperature, light/dark cycles and environmental barriers in the Institute of Biological Sciences (ICB, UFMG). For infections, 10,000 trypomastigotes were injected intraperitoneally. Mice condition and parasitemia were followed every 2–3 days by counting at least 25 fields under the microscope using the 40x objective lens. To determine parasite load in infected mouse tissues by qPCR, 30 days after the challenge, the animals were euthanized, their hearts were harvested and DNA extracted using the NucleoSpin tissue Kit (Macherey-Nagel) according to the manufacturer’s instructions. After DNA quantification with the Qubit 4 Fluorometer (Invitrogen), qPCR was performed using Power SYBR Green PCR Master Mix (Thermo Fisher Scientific) and primers targeting the *T*. *cruzi* satellite sequence (primer 1–5′-AST CGG CTG ATC GTT TTC GA-3′ (S = G or C) and primer 2–5′-AAT TCC TCC AAG CAG CGG ATA-3′). As an internal control, DNA amplification was also done with primers annealing within the mouse TNF gene (Forward primer 5’ CCCTCACACTCAGATCATCTTCT 3’ and reverse primer 5’ GCTACGACGTGGGCTACAG 3’). Reactions were performed in a final volume of 15 μL containing 100 ng of DNA template, the SYBR Green Master Mix and 10 μM of each primer. Standard curves were generated using serial dilutions of the target DNA, starting from 10000 pg—0.1 pg and parasite load was estimated as the number of parasites corresponding to 100ng of DNA PCR product. All procedures involving animals in this study were approved by the Animal Ethics committee of the Universidade Federal de Minas Gerais under protocol number 031/09. All care was taken to minimize animal suffering.

### Thin layer chromatography (TLC) and mass spectrometry analyses

The lipid extraction and TLC were performed based on Mina et al (2009) [36], but with slight modifications as briefly described below. For lipid extraction, 1 x 10^8^ *T*. *cruzi* epimastigotes, *L*. *major* promastigotes or Vero cells were collected by centrifugation (1000 x g, 8 min) and washed twice in PBS. The parasites (*T*. *cruzi* and *L*. *major*) were resuspended in 400 μL liver infusion tryptose (LIT) without FBS containing 5 μM NBD C_6_-ceramide (Thermo Scientific). and incubated for 2 h at 26°C (or at 24°C for *L*. *major*). Vero cells were resuspended in FBS-free RPMI containing 5 μM NBD C_6_-ceramide and incubated for 1h at 37°C. After washing in PBS twice to remove the excess of free ceramide, the cells were resuspended in 1.2 mL chloroform:methanol:water (1: 2:0.8 v/v), transferred to glass tubes and vortexed vigorously. The resulting extract was incubated on ice and vortexed every 5 min for 30 min. To continue the extraction, 312 μL of chloroform was added followed by vigorous agitation (vortex), and finally, 312 μL of water was added followed by vigorous agitation (vortex). The extracts were then centrifuged at 1200 x g (measure) for 10 minutes, and the lower phase transferred to a new glass tube and dried in vacuum centrifuge. The dried extracts were resuspended in 30 μL chloroform: methanol (1:1) and 5 μL were applied to a HPTLC silica plate (Merck) and separated using the eluent system chloroform:methanol:aqueous 0.25% KCL (55:45:10). TLC plates containing the NBD ceramide-labeled samples were visualized using a laser scanner (Typhoon FLA 9000 biomolecular imager).

For lipidomic mass spectrometry analyses, cell pellets with 10^9^ epimastigotes, were extracted in chloroform/methanol/water allowing the chloroform rich layer to be dried prior to electrospray mass spectrometry (ES-MS) analysis [37]. The lipid extracted was suspended in a 2:1 methanol/chloroform and analysed on a Thermo Exactive Orbitrap mass spectrometer by electrospray in both positive and negative ion modes. Samples were also analysed on an AB Sciex 4000 QTRAP, utilizing MS/MS both parent and daughter scanning modes using nitrogen as the collision gas, with collision energies between 35 and 55 V. Each spectrum encompasses at least 30 repetitive scans. Phospholipid species annotations were determined in reference to previous assignments [38] and the LIPID MAPS database.

### Drug sensitivity assays

Resazurin sodium salt (Sigma-Aldrich) was diluted in PBS 1X to a working solution of 2.5 mM and filter-sterilized before use. The initial parasite density was optimized as follows. 2-fold serial dilutions of log-phase culture ranging from 0.25 to 0.039 x 10^6^ parasites/well were seeded in 96-well plates in a final volume of 100 μL and incubated for 72 or 96 h at 28°C. After incubation time, 10 μL of resazurin solution was added to each sample and incubated, under light protection, at 28°C for 4 h. Emitted fluorescence was measured at Ex528/Em590, using a Varioskan LUX microplate reader (Thermo Scientific). Resultant data were analyzed using the software GraphPad Prism 8.0.1.

Tamoxifen (T5648, Sigma-Aldrich) and benznidazole were kindly provided by Silvia R. B. Uliana (University of São Paulo) and Silvane M. F. Murta (René Rachou Institute, FIOCRUZ), respectively. Tamoxifen (40 mM), benzazepanes (50 mM), and benznidazole (50 mM) were prepared in DMSO and used to prepare the 1:2 serial dilutions in 96-well plates with final volume of 50 μL/well. 1.25 x 10^5^ log-phase epimastigotes (50 μL) were added to 96-well plates and incubated at 28°C for 72 h. Each molecule was assayed in at least ten concentrations (250–0,48 μM) and the values standardised against the corresponding values obtained with DMSO. Cell viability was evaluated by the Alamar blue assay [39]. The half-maximal inhibitory concentration (IC_50_) of the dose-response curves was calculated using the sigmoidal dose-response curve (variable slope) using GraphPad Prism 8.0.1.

### Statistical analyses

Statistical analyses were performed using GraphPad Prism 8.0.1 (GraphPad Software, San Diego, California, USA) and STATA.MP 17 (StataCorp. 2021. Stata Statistical Software: Release 17). The reported values are means ± SD from n biological experiments, as indicated in the figure legends. The normality of the distribution of all experimental results was assessed using Shapiro-Wilk normality tests in conjunction with Q-Q plots and variance equality was analyzed using the Bartlett’ test. When the values were normally distributed, parametric tests were used for the analysis. Statistical significances of differences between the mean values of experimental groups were evaluated using the unpaired t-test for comparisons between two groups. One-way analysis of variance (ANOVA) was used for comparisons between more than two groups and when there is only one independent variable. If homogeneity of variance was not obtained for 2-way ANOVA, no further significance test was performed. For drug sensitivity assays, statistical differences between IC_50_ values were determined using the Kruskal-Wallis test. We acknowledge our statistical analyses requires further development particularly in the case of time series data.

## Results

### Topology, 3D structure predictions and gene expression analysis of *TcIPCS*

*TcIPCS* gene was previously identified as a functional ortholog of the fungal AUR1 gene [14,22]. In contrast to *T*. *brucei*, which has sequences encoding four distinct isoforms of sphingolipid synthases (SLS) [40], *T*. *cruzi* and *L*. *major* encode only one copy of the *IPCS* gene. Since the CL Brener clone of *T*. *cruzi* has a hybrid genome with two different haplotypes, named Esmeraldo-like and Non-Esmeraldo-like, sequences from the two alleles of the *TcIPCS* gene, each corresponding to one haplotype, were identified on chromosome 39 of this strain [22] (www.tritrypdb.org). The *TcIPCS* Esmeraldo-like (TcCLB.506885.124) and the Non-Esmeraldo-like (TcCLB.510729.290) alleles share 96% sequence identity at both the nucleotide and amino acid levels with both alleles encoding a protein with 335 amino acids. Genomic analyses based on the TriTrypDB (www.tritrypdb.org) also revealed that both *TcIPCS* alleles are syntenic with the *L*. *major IPCS*.

**Fig 1:**
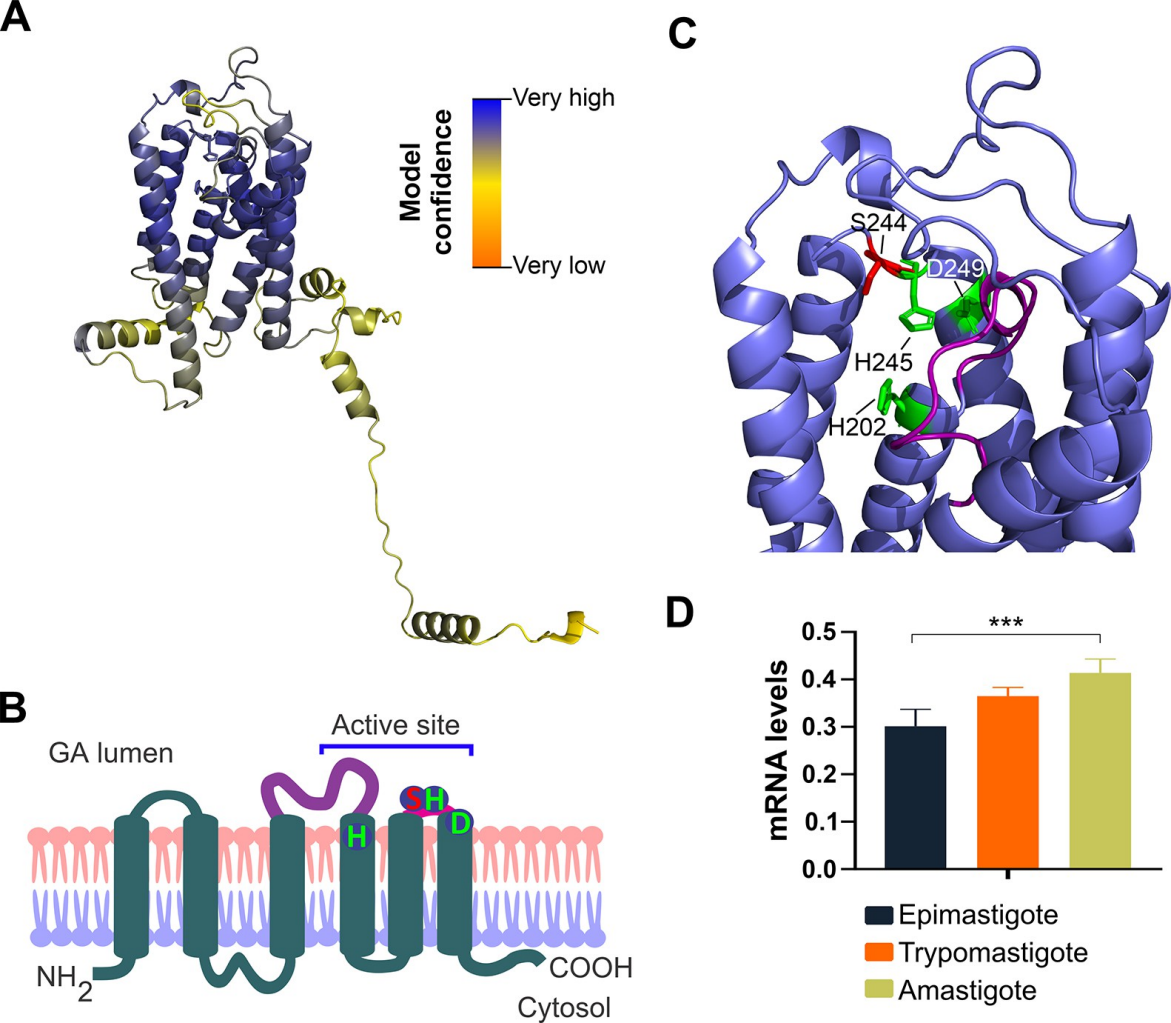
TcIPCS1 model prediction and topology. (A) Rosetta prediction of TcIPCS Esmeraldo-like allele (TcCLB.506885.124). Color palette indicates per-residue confidence score (pLDDT), which ranges from 0 to 100. (B) Topology diagram (schematic) of TcIPCS. (C) Key residues for the catalytic triad (H202, H245, D249) and substrate selectivity (S244). Predicted location of the TcIPCS active site with residues and loops are indicated. (D) Quantitative RT-PCR analysis of the *TcIPCS* transcript levels obtained from RNA isolated from amastigotes and tissue culture derived trypomastigote stages as well as epimastigote cultures. Statistical significance was determined by one-way ANOVA (n = 5; ***, *P* < 0.001).

Due to their properties as transmembrane proteins, no crystal structure has been determined for IPCS or for any other protein belonging to the SLS family. In agreement with the results from previous topology analyses [40,41], the predicted 3D structure of TcIPCS showed that the enzyme is folded into six alpha helices that correspond to six transmembrane domains (Fig 1A and 1B). The model also predicts the existence of an unstructured C-terminal region of 70 amino acids facing the cytosol, which may play a regulatory role, perhaps as a site of interaction with other proteins. According to the 3D model and the expected IPCS subcellular localization [14,24,42], the catalytic triad (H202-H245-D249) [40] is expected to be partially buried on what would be the exoplasmic leaflet of the Golgi apparatus membrane, forming a binding pocket-like structure (Fig 1C). The S244 residue, which have been found to determine phospholipid head group selectivity in sphingomyelin synthases in *T*. *brucei* [43] and in the human enzyme [24], was found positioned above the catalytic triad, possibly restricting substrate interaction.

Based on primary sequence identity, orthologues of the yeast *IPCS* gene were identified in different *Leishmania* species and in *Trypanosoma brucei* [14,44]. Among the four orthologue genes identified in *T*. *brucei*, *Tb*SLS1 encodes an inositol phosphorylceramide (IPC) synthase, whilst *Tb*SLS4 encodes a novel bi-functional enzyme with the ability to catalyze the biosynthesis of both IPC and SM. The alignment of the sequences from the single gene present in *L*. *major* and the *T*. *brucei Tb*SLS1 enzymes showed that they share 52% and 49% amino acid identity, respectively, with the *T*. *cruzi* IPCS (S1 Fig). Despite a low level of overall primary sequence identity, these proteins contain not only highly conserved motifs corresponding to the catalytic triad and the serine involved in substrate selectivity, but they are also structurally conserved, as showed by the alignment of the predicted models corresponding to *Lm*IPCS, *TbSLS1* and TcIPCS (S1 Fig).

Quantitative RT-PCR data generated from RNA isolated from epimastigotes, tissue culture derived trypomastigotes (TCT) and amastigotes indicated that the *TcIPCS* gene is constitutively expressed in all stages of the parasite life cycle (Fig 1D). Despite a small increase in transcript levels observed in amastigotes compared to epimastigotes, these results are in agreement with a previously published transcriptome dataset obtained from *in vitro* cultured epimastigotes, intracellular amastigotes and tissue culture-derived trypomastigotes [27]. These results are also consistent with previously published data showing that all forms of *T*. *cruzi* express IPC synthase activity [45].

### Generation of *T*. *cruzi IPCS* knockout parasites

**Fig 2:**
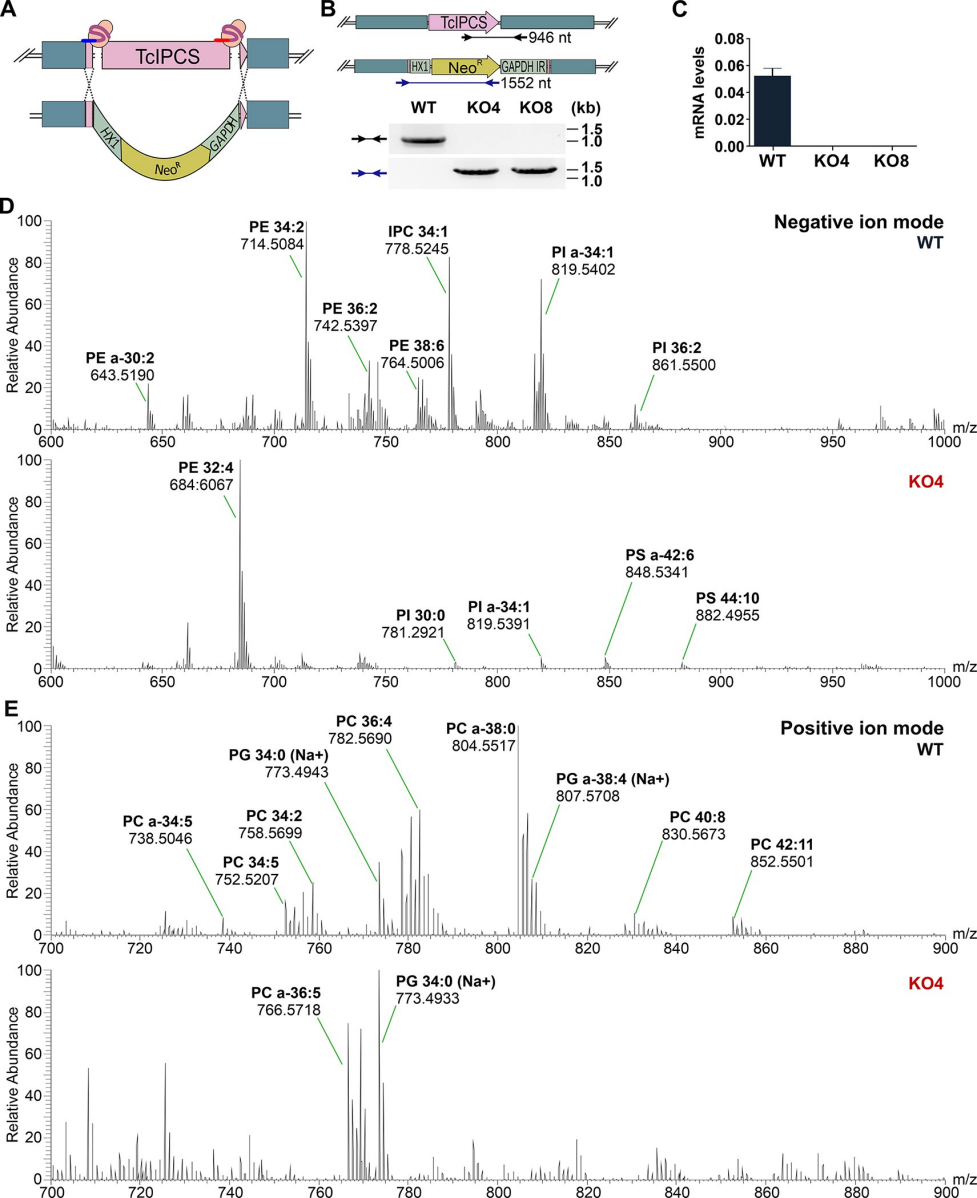
CRISPR-Cas9 strategy to generate *TcIPCS* knockout in *T*. *cruzi* epimastigotes. (A) Schematic representation of the Cas9 ribonucleoprotein complexes with the two sgRNAs and their annealing position in the *TcIPCS* locus. A DNA fragment containing the *TcIPCS* homologous sequences flanking a Neomycin resistance cassette to allow homologous recombination repair is also shown. After epimastigote transfection, replacement of the *TcIPCS* coding region by the Neomycin resistance gene is indicated. (B) PCR analysis of wild type and *TcIPCS* knockout epimastigotes. The annealing position of primers are shown in the schematic representation of one wild type and one disrupted *TcIPCS* allele with the predicted sizes for the amplicons indicated below the figure. Agarose gel electrophoresis analyses of PCR products obtained with DNA extracted from WT epimastigotes and from two *TcIPCS* knockout (KO) cloned cell lines, KO4 and KO8 and the indicated primer sets. (C) Relative expression levels of *TcIPCS* transcripts quantified by RT-qPCR using RNA extracted from WT and two *TcIPCS* KO cell lines, KO4 and KO8. (D) Negative ion ES-MS lipidomic analysis of WT and TcIPCS KO4 lipid extracts. Spectra show survey scans (600-1000m/z) of WT (upper figure) and KO4 epimastigotes (bottom figure) showing a heterogeneous mixture of PI, IPC, PS and PE. (E) Positive ion ES-MS lipidomic analysis of WT and TcIPCS KO4 lipid extracts. Spectra show survey scans (600-1000m/z), of WT (upper figure) and KO4 epimastigotes (bottom), showing a mixture of PC and PG species.

Using a CRISPR-Cas9 protocol based on the transfection of epimastigotes with purified recombinant *Staphylococcus aureus* Cas9 (rSaCas) complexed with *in vitro* transcribed single-guided RNAs (sgRNA), we were able to generate two cloned cell lines in which both alleles encoding the *T*. *cruzi IPCS* were disrupted. The two sgRNAs have sequences complementary to the 5’ and 3’ ends of the coding region of both *TcIPCS* alleles and direct the DNA cleavage by the Cas nuclease at both sites [28]. A DNA fragment containing the Neomycin resistance gene (*Neo*^*R*^) flanked by 5’ and 3’ untranslated regions (UTR) plus intergenic sequences from two *T*. *cruzi* genes (*HX1* and *GAPDH*) inserted downstream and upstream from sequences of the *TcIPCS* coding was used as a DNA template for homology directed repair (HDR) of the double strand break (Figs 2A and S2). After transfecting epimastigotes with the ribonucleoprotein complex together with the HDR DNA donor fragment, the transfected population was submitted to G418 selection, cloned by serial dilution and genotyped using PCR and different primer sets (Fig 2B). PCR analyses from DNA extracted from two cloned, G418 resistant cell lines confirmed the deletion of the *TcIPCS* sequence and the insertion of the *Neo*^*R*^ gene (Fig 2B). *TcIPCS* gene knockout was further confirmed by mRNA amplification and quantitative RT-PCR analyses, which showed no amplification products with RNA extracted from the two *TcIPCS* knockout cell lines (hereinafter referred to as clones KO4 and KO8). In contrast, as shown before, RT-PCR products of the *TcIPCS* mRNA were generated with RNA extracted from wild type (WT) epimastigotes (Fig 2C). These results confirmed the generation of viable *T*. *cruzi* epimastigotes with both *TcIPCS* alleles disrupted.

To further confirm the deletion of the *TcIPCS* gene in the knockout cell lines, we compared the lipid profiles of wild type and knockout parasites obtained using electrospray ionization mass spectrometry (ESI-MS). As shown in Fig 2D (upper panel) several lipids were identified in the extracts from WT epimastigotes including several PE and PI species as well as the peak at m/z 778.6 identified as the main IPC species with a 34:1 ceramide moiety. This IPC species is absent in the *TcIPCS* KO4 epimastigotes (Fig 2D, upper panel) supporting the claim that the IPCS gene has been deleted. Notably, in addition to the loss of IPC, there are other significant changes in the lipid content of the *TcIPCS* KO4 epimastigotes. One might expect there to be significant increase in the amount of unused PI, the precursor to form IPC. However, the knockout cells seem to have moderated their production of PI but dramatically increased the production of phosphatidylthalonamine (PE, 32:4) as well as phosphotidylserine (PS) species containing very long polyunsaturated fatty acids (Figs 2E and S2). We also investigated the positive ion mode to look for phophotidylcholine (PC) species in both WT and KO4 cells (Fig 2E). As expected, WT epimastigotes showed a range of diacyl and acylalkenyl species. However, rather surprisingly, the TcIPCS KO4 cells also showed significant alterations in the PC profile compared to WT cells.

### Characterization of growth, differentiation and *in vitro* infection capacity of *TcIPCS* null mutants

**Fig 3:**
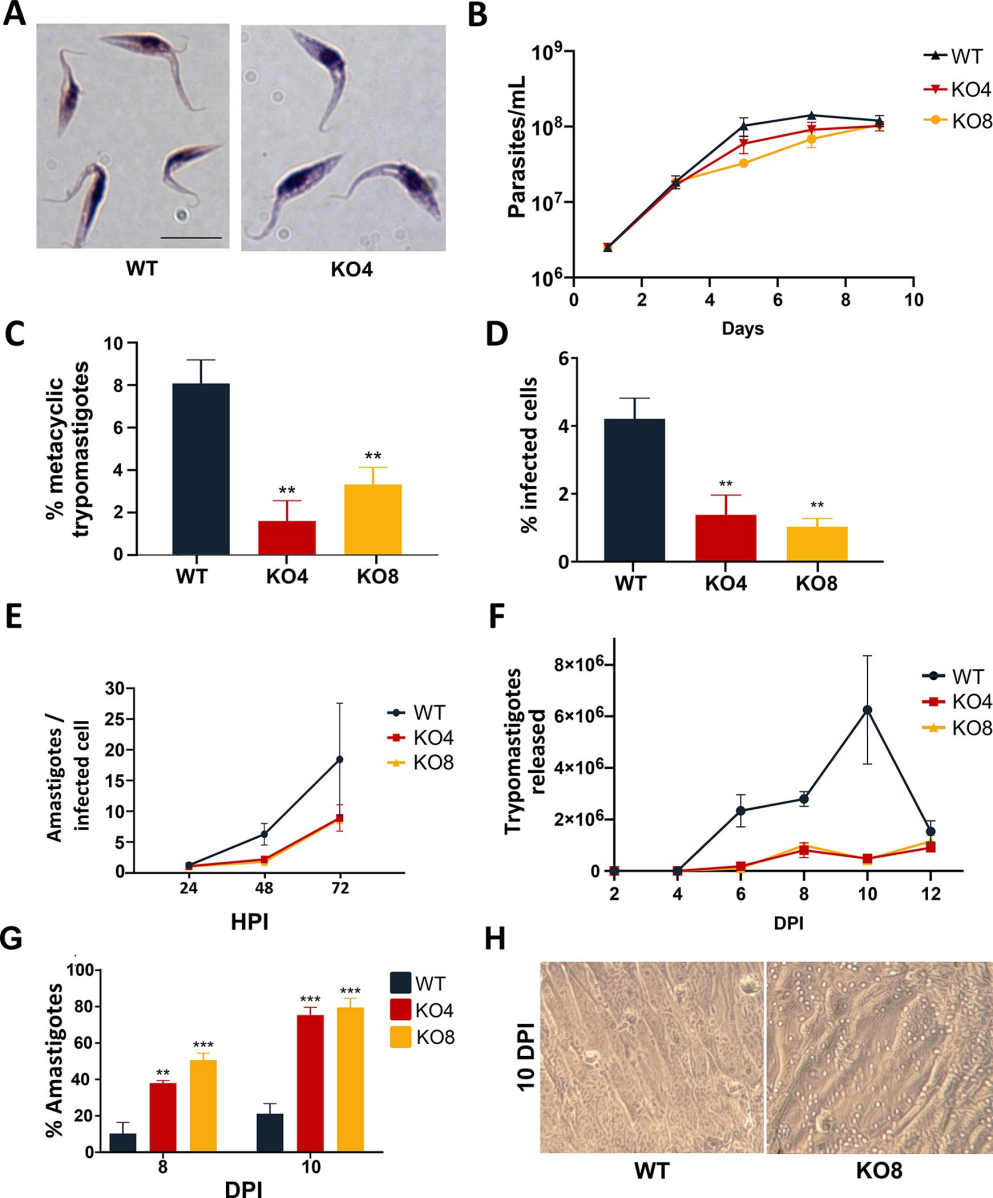
Metacyclogenesis and infection capacity of *TcIPCS* knockout mutants. (A) Analyses by light microscopy of WT epimastigotes and one *TcIPCS* KO cell line (KO4) cultured in LIT medium. *Bar* = 10 μm. (B) Growth curve of epimastigote cultures from WT and two *TcIPCS* knockout cell lines, KO4 and KO8. Parasites were diluted in LIT medium to a concentration of 2.5 x10^6^ cells/mL and parasite numbers were determined over 9 days. Statistical significance could not be established for the analysis shown in B as described in Methods. (C) Percentages of metacyclic trypomastigotes on day 9 of cultures of WT and the two cloned *TcIPCS* KO cell lines (KO4 and KO8). Statistical significance was analyzed by one-way ANOVA (n = 3; ***, *P* < 0.001). (D-G) *In vitro* infection of Vero cells with tissue culture-derived trypomastigotes from WT and the *TcIPCS* KO cell lines KO4 and KO8. The percentages of infected cells at 24 hours post-infection are shown in D; the numbers of intracellular amastigotes per infected cell are shown in E; the numbers of trypomastigotes released in the supernatant of infected cells at different time points post-infection are shown in F. The percentages of amastigotes present in the supernatants of cultures on days 8 and 10 post-infection as well as their morphology are shown in G and H, respectively. Multiplicity of infection (MOI) was 10 parasite per cell. Statistical significance was analyzed by one-way ANOVA for data shown in D and G (**P < 0.01, *** P < 0.001). Statistical significance could not be established for the analyses shown in E and F as described in Methods.

To assess the impact of the *TcIPCS* deletion on the growth of epimastigotes, the two G418 resistant *TcIPCS* KO cell lines were diluted in fresh media and parasite numbers were determined over 10 days. As shown in Fig 3A and 3B, despite the significant changes in the phospholipid content of the parasite membranes, there were no significant differences in the morphology of epimastigotes and only a small decrease in parasite numbers in the late log phase of the growth curves (day 7) of the *TcIPCS* KO8 mutant compared to WT epimastigotes. To establish if the *TcIPCS* deletion affects metacyclogenesis, i.e., the differentiation from epimastigotes to metacyclic trypomastigotes, we determined the percentage of metacyclic trypomastigotes on day 9 of culture of WT parasites and *TcIPCS* KO mutants. As described previously [43], when cultivated in LIT medium, epimastigotes of the CL Brener strain begin to differentiate into metacyclic trypomastigotes on day 9, when about 8–10% of epimastigotes are transformed into infective, metacyclic trypomastigotes *in vitro*. Compared to WT parasites, both *TcIPCS* KO mutants displayed a significant decrease in the percentage of metacyclic trypomastigotes that were generated on day 9 of culture (Fig 3C).

Despite the reduced numbers of metacyclic trypomastigotes obtained in the stationary phase of the growth curve of *TcIPCS* KO mutants in LIT medium, we were still able to infect Vero cells with both WT and *TcIPCS* mutants. After infection, we harvested enough tissue culture-derived trypomastigotes (TCTs) to perform *in vitro* infection assays using the different cell lines with a MOI 10:1. In vitro infection assays showed a significant reduction in the numbers of cells infected with TcIPCS KO mutants compared with cells infected with WT parasites (Fig 3D), however reductions in the numbers of intracellular amastigotes could not be significantly determined with current statistical methods (Fig 3E). Albeit further statistical development is also required to demonstrate significance, the numbers of trypomastigotes released in the culture supernatant over time appears to be reduced in cultures infected with TcIPCS KO mutants compared to WT (Fig 3F). These results demonstrated that the infection capacity of TcIPCS KO trypomastigotes was affected. Furthermore, it may be possible that the replication rates of intracellular amastigotes as well as their capacity to differentiate into trypomastigotes are also impaired in the TcIPCS. Interestingly, culture supernatants of cells infected with the *TcIPCS* KO mutants presented a large number of amastigote-like forms, constituting up to 80% of all parasite forms in the supernatant on day 10 post-infection, compared to 20% in the supernatant of cells infected with WT parasites (Fig 3G and 3H). This result suggests that most trypomastigotes from mutant parasites that are released from the infected cells differentiated into extracellular amastigotes. Transformation of extracellular TCT into amastigote-like forms has been described to occur *in vivo* and *in vitro*, under conditions of low pH or after treatment of trypomastigotes with phosphatidylinositol-specific phospholipase C (PI-PLC) [46]. Here, we observed increased differentiation from extracellular trypomastigotes into amastigote-like forms in the culture supernatant of cells infected with the *TcIPCS* KO mutants. Altogether, our data showed that the deletion of *TcIPCS* gene interferes with the parasite capacity to infect and multiply within host cells as well as with the differentiation process that occurs both in the vector and in the mammalian host.

### Sensitivity of the *TcIPCS* null mutants to inhibitors of IPCS and other compounds with antiprotozoal activity

**Fig 4:**
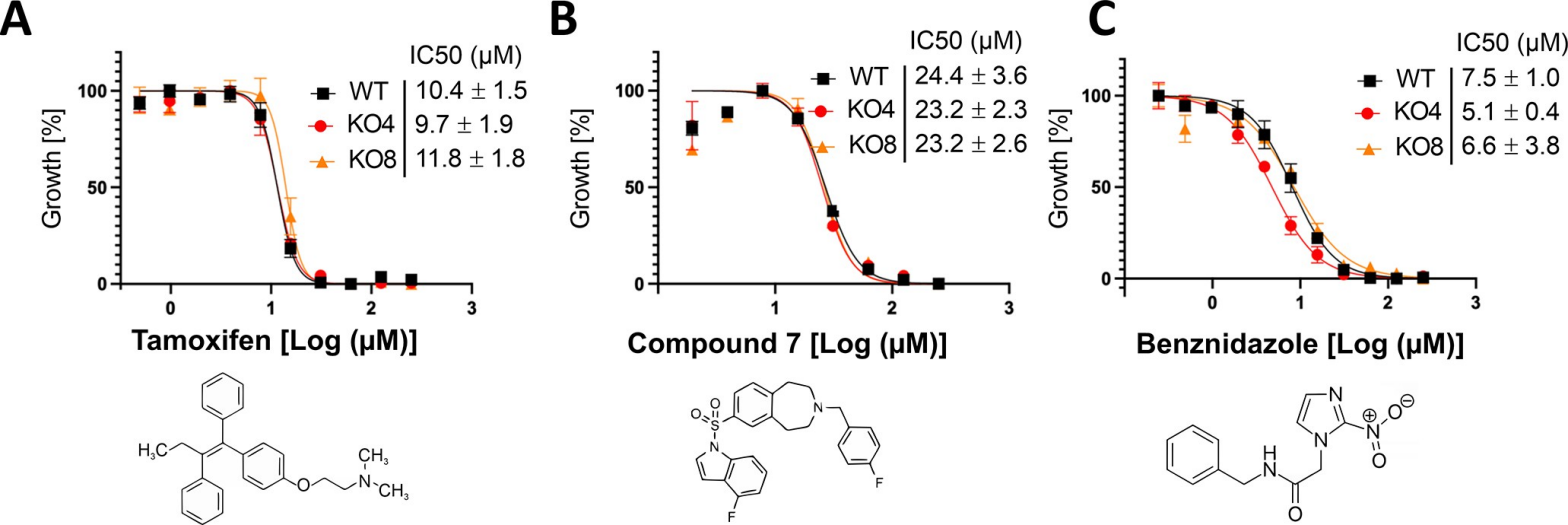
Sensitivity of epimastigotes to inhibitors of *Leishmania* IPCS. Epimastigotes cultures of WT and *TcIPCS* KO cell lines KO4 and KO8 were incubated in the presence of increasing concentrations of tamoxifen (A), benzazepane compound 7 (B) and benznidazole (C). Viability of the cells were determined by Alamar blue assay. Data points are mean values ± SD of three determinations. Representative experiments performed in triplicates are shown. IC_50_ values were determined as the mean ± SD of at least 3 independent biological replicates. There were no statistically significant differences between IC_50_ values, as determined by the Kruskal-Wallis test.

Several compounds have been evaluated for their activity against fungal and protozoan IPCS. To verify whether disruption of the *TcIPCS* gene and, consequently, the biosynthesis of IPC may affect the parasite susceptibility to these drugs, we determined the half-maximal inhibitory concentration (IC_50_) of epimastigotes from WT strain and the two *TcIPCS* KO cell lines for two classes of compounds. Tamoxifen is an oral drug that has been in use for many years to treat breast cancer. Previous work has shown that this molecule is also active against *T*. *cruzi* [47] and several species of *Leishmania* [48] and may have IPCS as a target. Recent experiments using membrane microsomal extracts containing the *L*. *major* IPC synthase preparations indicated that tamoxifen inhibits the *Leishmania* IPCS with an IC_50_ value of 8.48 μM [48]. To verify whether TcIPCS is a target of tamoxifen, we tested this compound as a growth inhibitor of epimastigotes from WT and from the two *TcIPCS* KO cell lines. As shown in Fig 4A, no difference in the sensitivity was observed between the three parasite lines, indicating that the presence of IPCS activity does not correlate with the effectiveness of this compound against *T*. *cruzi*.

We also tested a group of compounds named benzazepanes, which have been identified as inhibitors of the *L*. *major* IPCS enzyme at nanomolar concentrations [21]. As shown in Fig 4B, WT epimastigotes displayed a moderate sensitivity to the benzazepane compound 7, with an IC_50_ (24 μM) significantly higher compared to *L*. *major* (which showed an IC_50_ of 5.5 μM) [6]. Moreover, no differences in epimastigote growth inhibition were observed when the *T*. *cruzi* WT strain was compared with the two *TcIPCS* KO cell lines, or when other classes of benzazepane compounds were tested (S3 Fig). Thus, similar to the results observed with tamoxifen, and distinct from the *Leishmania* enzyme, dose response assays with the benzazepanes suggest that *T*. *cruzi* IPCS is not a target for these compounds.

Benznidazole (BZ) is one of the two drugs available for the treatment of Chagas disease, but significant differences in drug susceptibility among *T*. *cruzi* strains have been reported [6]. As a control, we have also determined the IC_50_ values for benznidazole and showed that they are similar to that previously described for the CL Brener strain [49]. In addition, no significant differences in the sensitivity to BZ were observed when the three parasite lines were compared after exposure for 72 h to different concentrations of this compound (Fig 4C).

### IPC production in *Tc*IPCS null mutants and addback cell lines

**Fig 5:**
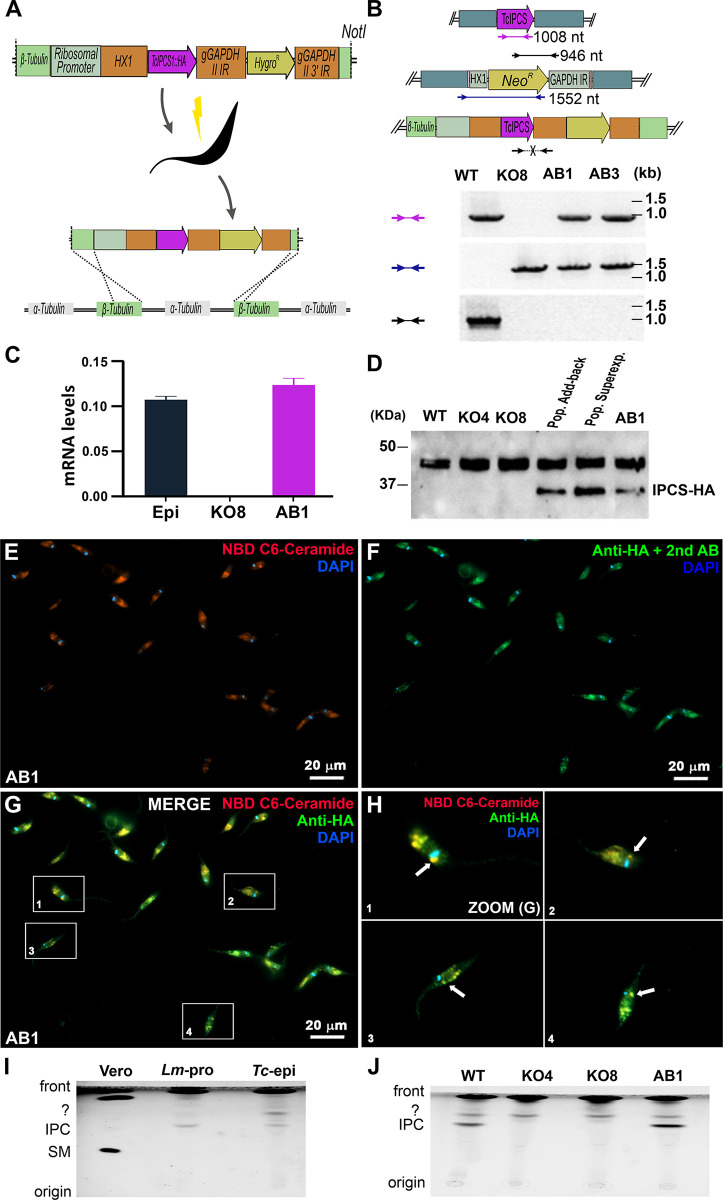
Generation of *TcIPCS* addback cell lines, protein localization and enzyme activity. (A) Schematic representation of the linearized *TcIPCS-HA* expression vector used for transfection and integration in the *tubulin* locus to generate the addback line. (B) PCR analysis of WT, *TcIPCS* KO cell line (KO8) and two cloned addback cell lines (AB1 and AB3) obtained after transfection of the *TcIPCS* KO8 clone with the *TcIPCS-HA* expression vector. Annealing positions of the primers and the expected sizes of the amplicons are indicated in the schematic representation of one wild type *TcIPCS* allele (top figure), one disrupted *TcIPCS* allele (medium) and in the addback sequence inserted in the *tubulin* locus (bottom). Agarose gel electrophoresis analyses showed PCR products obtained with DNA extracted from WT epimastigotes, the *TcIPCS* KO8 cell lines and the two cloned addback cell lines AB1 and AB3. (C) Relative expression levels of *TcIPCS* transcripts quantified by RT-qPCR using RNA extracted from WT, from the Tc*IPCS* KO cell line KO8 and from the addback cell line AB1. (D) Western blot analysis with anti-HA antibody of HA-tagged *TcIPCS* and total protein extracts from WT, *TcIPCS* KO cell lines KO4 and KO8, a transfected population derived from the KO8 cell line (pop. addback) and a transfected population derived from WT epimastigotes (pop super-expressor). The bands corresponding to the HA-tagged TcIPCS protein with a MW of 37 KDa are indicated. (E-H) Fluorescence microscopy analyses of epimastigotes incubated with NBD C_6_-Ceramide to stain ceramide rich regions (red), such as the Golgi apparatus (E). Parasites showing the immunostaining using anti-HA antibody (green) indicate TclPCS protein localization (F). Merged image showing the co-localization of the protein with ceramide-rich regions, close to the kinetoplast, indicates that TcIPCS localizes to the Golgi apparatus (G). White arrows indicate co-localization of the TclPCS protein with NBD C_6_-ceramide close, but not at the same location to the parasite kinetoplast, where the Golgi apparatus is localized (H). (I) HP-TLC analysis of IPC present in extracts from *T*. *cruzi* epimastigotes, Vero cells (negative control) and *L*. *major* (positive control). (J) HP-TLC analysis of IPC present in *T*. *cruzi* epimastigotes extracts derived from WT, *TcIPCS* KO4 and KO8 cell lines as well as in the *TcIPCS* addback cell line AB1. In I and J, cells were incubated in presence of fluorescent ceramide and immediately used for preparation of lipid extracts. The migration positions of fluorescent inositol phosphorylceramide (IPC), sphingomyelin (SM) as well as the origin (O) and the front (F) are indicated. The presence of an unknown putative lipid was also identified.

**Fig 6:**
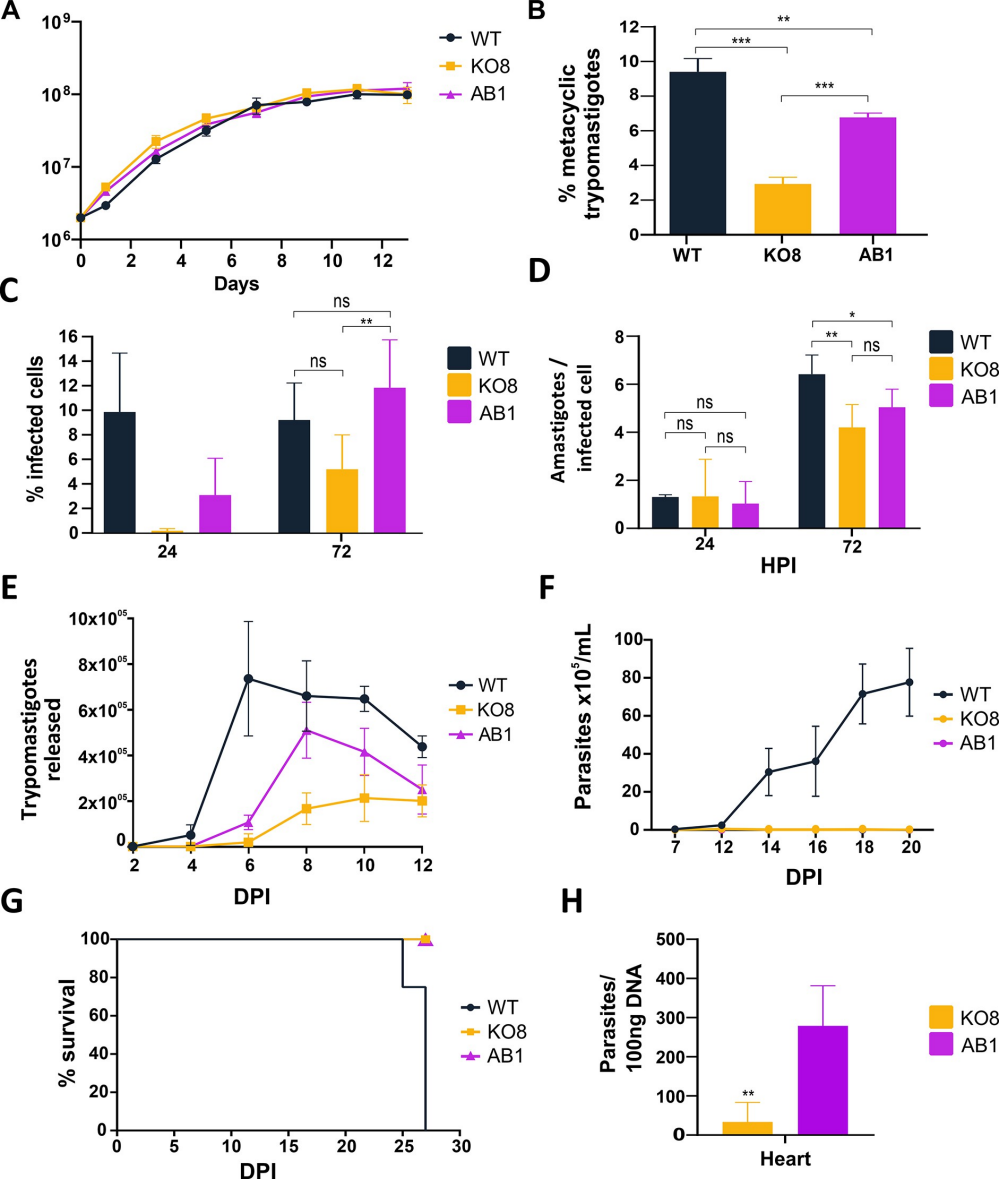
Metacyclogenesis and infection capacity of *TcIPCS* knockout mutants and addback cell lines. (A) Growth curve of WT, *TcIPCS* knockout (KO8) and *TcIPCS* addback (AB1) parasites. Parasites were diluted to a concentration of 2.0 x10^6^ parasite/mL, and parasite density was determined over 13 days. Statistical significance could not be established for A as described in Methods. (B) Percentages of metacyclic trypomastigotes on day 9 of cultures of WT, one cloned *TcIPCS* KO cell line (KO8) and one *TcIPCS* addback cell line (AB1) in LIT medium. Statistical significance was analyzed by one-way ANOVA (n = 3; **, *P* < 0.01, ***, *P* < 0.001). (C-E) *In vitro* infection of Vero cells with tissue culture-derived trypomastigotes from WT, one cloned *TcIPCS* KO cell line (KO8) and one *TcIPCS* addback cell line (AB1). The percentages of infected cells (C) and the numbers of intracellular amastigotes per infected cell (D) at 24- and 72-hours post-infection are shown; the numbers of trypomastigotes released in the supernatant of infected cells at different time points post-infection are shown in E. Multiplicity of infection (MOI) was 10 parasite per cell. Statistical significance was analyzed by one-way ANOVA in C and D, but data from E could not be statistically assessed within the current ANOVA, as described in Methods. (F-G) Interferon gamma knockout mice were infected with 10,000 culture-derived trypomastigotes of the WT, *TcIPCS*-KO (KO8) and *TcIPCS* addback (AB1). Parasitemia (F) and survival rates (G) were followed for 20- and 30-days post infection (DPI). Data points are mean values ± SD from four mice per group. Data from F and G could not be statistically assessed. (H) Thirty days after challenge infection, IFN-g KO mice were sacrificed, their hearts were harvested, and DNA extracted. The number of parasites was determined by quantitative PCR (qPCR). Statistical significance was analyzed by two-tailed unpaired *t-*test (n = 5; ***, *P* <0.001).

To further investigate the role of IPC during infection, we generated *TcIPCS* addback parasites by transfecting one of the T*cIPCS* KO mutant cell lines (KO8) with the *T*. *cruzi* expression vector pROCK-Hygromycin [30] containing sequences of the full length, HA-tagged *TcIPCS* coding region. As described in Fig 5A, transfection of epimastigotes with the pROCK vector linearized with NotI allowed homologous recombination within the *tubulin* locus [30]. This plasmid has been used in several studies for the ectopic expression of genes in *T*. *cruzi* under the control of the rRNA promoter. After transfection and selection of a resistant parasite population with hygromycin and neomycin, two cloned cell lines were generated and DNA was extracted from both clones. At the same time, we transfected wild type epimastigotes with the same linearized vector and selected an hygromycin resistant population. PCR analyses with different primer combinations demonstrated that the tagged *TcIPCS* coding sequence was inserted in the genomes of the two addback cell lines as well as in the transfected WT epimastigotes (Fig 5B). RT-qPCR showed that *TcIPCS* mRNA was expressed in similar levels in WT and in the transfected addback cell line (Fig 5C). Western blot analyses with anti-HA antibody also showed a 37 KDa protein corresponding to the HA tagged *TcIPCS* present in the extracts of the transfected addback population as well as in the addback cell line (Fig 5D). Similarly, extracts from the transfected, hygromycin resistant population of WT epimastigotes also showed a 37 KDa tagged *TcIPCS* protein. As expected, the 37 KDa band was not observed either in protein extracts from WT parasites or in the two *TcIPCS* knockout cell lines (Fig 5D). A 45 KDa band identified in all protein extracts is likely due to non-specific binding of the anti-HA antibody, as previously observed (Aprigio-Santos, personal communication). We have also showed that epimastigotes of the *TcIPCS* addback cell line have a growth curve similar to WT and *Tc*IPCS KO mutant (Fig 6A).

Using the HA-tagged *TcIPCS* addback cell line, we evaluated the cellular localization of TcIPCS protein in epimastigotes by performing immunofluorescence (IF) assays with an anti-HA antibody. Microscopy analysis showed that only parasites containing HA-tagged TclPCS (AB1) were labelled (Figs 5E–5H and S4). The specific intracellular labelling clearly showed that the protein was associated with the kinetoplast, in close proximity to the Golgi apparatus and is also localized at the opposite end of the parasite. To confirm that TcIPCS indeed co-localizes with the Golgi apparatus, we labelled parasites with NBD C_6-_ceramide, a probe that accumulates in ceramide-rich regions of parasites and mammalian cells. As shown in Figs 5E and S5, incorporation of ceramide in WT, KO8 and AB1 epimastigotes revealed a similar labelling pattern in all parasites. As shown previously, the ceramide probe clearly stains the Golgi apparatus, which can be identified as close to the kinetoplasts. Additionally, round-shaped structures at the opposite end of the parasite were also labelled by the ceramide probe in a pattern that strongly suggests the labelling of reservosomes, organelles rich in different types of lipids including ceramides that are typically present in epimastigotes [50,51]. Our results confirmed that both ceramide-rich regions (Fig 5E) and HA-tagged TclPCS (Fig 5F) present strong co-localization (Fig 5G and 5H, white arrows), thus corroborating the subcellular localization of the TclPCS protein in the Golgi apparatus. Additionally, co-localization with the ceramide at the opposite end of the parasite, indicates the presence of the TcIPCS protein also in reservosomes.

To verify whether the knockout mutants have lost their ability to produce IPC and that this enzymatic reaction can be restored by the ectopic expression of the HA-tagged *TcIPCS* gene in the addback cell lines, we performed metabolic labelling assays using fluorescent NBD C_6_-ceramide as substrate. As a positive control, we used *L*. *major* promastigotes that have been previously shown to incorporate fluorescent ceramide into IPC [14]. We also incubated Vero cells with the fluorescent ceramide as a negative control since IPC is not synthesized in mammalian cells. Following fractionation by thin layer chromatography (TLC) it was revealed that both *L*. *major* promastigotes and *T*. *cruzi* WT epimastigotes produced fluorescent products with the same mobility, compatible with IPC. Neither the two *TcIPCS* knockout cell lines nor Vero cells showed an equivalently labelled lipid species (Fig 5I–5J). As shown before, incubation of Vero cells with fluorescent ceramide resulted in the production of sphingomyelin [14]. TLC analyses also revealed that the ability to convert fluorescent ceramide into labelled IPC was restored in the addback cell line, demonstrating that the HA-tagged enzyme is active in this cell line.

### Rescuing IPCS activity partially restores parasite infectivity

IPCS addback cell lines, generated by ectopic expression of the *TcIPCS* gene in the knockout mutant Tc*IPCS* KO8, were used to investigate whether the restoration of IPCS activity was sufficient to rescue the knockout phenotype observed in the metacyclogenesis assay and during infection. As shown in Fig 6A, the addback cell line AB1 presents a similar growth curve compared to WT and *TcIPCS* KO8 epimastigotes. Although the rates of metacyclogenesis of the addback cell line was not fully rescued when compared with WT parasites, restoration of IPCS activity in the addback cell line resulted in a significant increase in the percentages of metacyclic trypomastigotes observed on day 9 in culture compared to the Tc*IPCS* KO8, a result that confirms the essential role of IPC during metacyclogenesis (Fig 6B). When equal amounts of trypomastigotes from WT and the addback cell line AB1 were collected from Vero cells and used to re-infect Vero cells at a MOI of 10:1, no significant differences in the percentages of infected cells were observed between infection with WT and addback parasites at 72 hpi. Although the difference in infection rates between WT and the KO8 mutant was not statistically significant, at 72 hpi, the percentage of cells infected with the addback cell line was significantly higher compared to cells infected with TcIPCS KO8 (Fig 6C). As shown before, a small difference in the numbers of intracellular amastigotes in cells infected with the KO8 mutant compared with WT parasites was observed. Re-expression of IPCS resulted in numbers of intracellular amastigotes that were not statistically different from amastigote numbers in cells infected with the mutant (Fig 6D). Finally, we also determined that the numbers of trypomastigote released in the supernatant of infected cultures appeared to be partially restored in the addback cell line, particularly at day 8 post-infection. A putative difference was observed between the addback cell line and TcIPCS KO8 and only a smaller putative difference was observed when the infection with WT and addback cell line were compared (Fig 6E). Thus, re-expression of TcIPCS appears to restore production of IPC in the mutants and there is a possible partial restoration in the capacity of the mutants to establish infection in vitro.

To verify whether the results obtained during *in vitro* infections, were also observed *in vivo*, we inoculated mice with trypomastigotes derived from WT, *TcIPCS* KO8 and the addback cell line AB1 and followed parasitemia for several days. Because infection of BALB/c mice did not result in detectable parasitemia even with WT parasites, we inoculated three groups of IFN-γ KO mice. As shown before [52], infection of IFN-γ KO mice with WT parasites of the CL Brener strain results in high parasitemia and 100% mortality approximately at day 25 (Fig 6F–6G). Similar to the results observed with *in vitro* infections, IFN-γ KO mice infected with the *TcIPCS* KO8 mutant cell line showed no parasitemia and 100% survival even at 20 days post-infection compared to WT parasites. Although no difference was observed in parasitemia between *TcIPCS* KO8 and the addback cell line AB1, since infection with both cell lines resulted in undetectable levels of parasitemia, qPCR analyses showed significant difference in tissue parasitism when mice infected with TcIPCS KO8 and addback cell lines were compared. As shown in Fig 6H, qPCR with DNA extracted from hearts from animals that survived 30 days post-infection (only animals infected with Tc*IPCS* KO8 and addback parasites survived past 27 days) revealed increased amount of parasite DNA in heart tissue of animals infected with addback compared to *TcIPCS* KO8. These results indicate that despite being able to infect and multiply within host cells *in vitro*, and to survive in the tissues of infected animals, the addback cell line has decreased ability to survive within the host bloodstream. The fact that we did not detect addback parasites in the bloodstream was not due to reduced expression of the TcIPCS in trypomastigotes, since western blot analyses of total cell extracts obtained from TCTs used to infect mice showed, as expected, that the addback copy of the *TcIPCS* gene is constitutively expressed in epimastigotes as well as in trypomastigotes (S6 Fig). It is more likely that, similar to *TcIPCS* KO parasites, the reduced capacity of the addback cell line to differentiate from amastigotes into trypomastigotes affects their ability to survive in the bloodstream.

## Discussion

Due to the non-mammalian nature of the complex sphingolipid IPC and its likely key role as a component of plasma membrane, the enzyme that catalyzes its formation (IPCS) has been considered as essential gene in the parasitic protozoa. That appears to be the case in *T*. *brucei*, where RNAi silencing of *TbSLS*1-4 resulted in growth arrest and cell death of bloodstream form trypanosomes [36]. In contrast, deletion of the gene encoding the *L*. *major* serine palmitoyltransferase subunit 2 (LCB2), the catalytic subunit of the first and rate limiting enzyme in the IPC biosynthetic pathway, showed that *de novo* sphingolipid biosynthesis is essential for differentiation but not for growth of *Leishmania* promastigotes [53]. Moreover, more recent work has shown that knockout of the LCB2 gene in *Leishmania* species is facilitated by compensatory deletion of a gene encoding a putative sterol transporter [54]. Surprisingly, it was also demonstrated that *L*. *major IPCS* null mutants are not only viable *in vitro* but acquire a highly virulent phenotype in mouse infections [55]. Here we showed that, in contrast to *T*. *brucei*, the *T*. *cruzi* IPCS, although essential for complex sphingolipid biosynthesis is, similarly to the *L*. *major* IPCS, non-essential for parasite viability. Our data show that, although *T*. *cruzi IPCS* KO epimastigotes are not able to incorporate fluorescent ceramide into IPC, and *TcIPCS* KO does not display a MS lipidomic profile in which IPC can be detected, the growth curve of epimastigotes lacking this sphingolipid may differ very little from the growth curve of WT epimastigotes. In contrast, the capacity of *TcIPCS* KO epimastigotes to differentiate into metacyclic trypomastigotes was significantly affected as well as the parasite’s capacity to differentiate from amastigotes into trypomastigotes which appears to be reduced in cultures infected with the mutants compared to WT. Moreover, the expression of an ectopic copy of *TcIPCS* and the consequent restoration of enzymatic activity partially rescued most of these phenotypes including the metacyclogenesis capacity and the capacity to differentiate from amastigote to trypomastigote after cell infection *in vitro*. In accordance with previous studies that have shown major differences in the phosphatidylinositol moiety of GPI-anchored mucins present in epimastigotes, which contain mainly 1-O-hexadecyl-2-O-hexadecanoylphosphatidylinositol and in metacyclic trypomastigotes, which contain mostly inositolphosphoceramide (IPC) [20], the absence of TcIPCS activity, and so inositolphosphoceramides would affect epimastigotes to trypomastigotes differentiation. The effect of the absence of IPC on amastigote differentiation to trypomastigotes, which occurs during *in vitro* infection of fibroblasts, could be also due to major changes in the surface components of parasite. A detailed characterization of the surface components in wild type, *TcIPCS* KO and addback parasites is underway and will prove crucial for furthering the understanding of the role of IPC in infection and disease.

Notably, unlike the situation reported for *L*. *major*, ablation of *TcIPCS* rendered *T*. *cruzi* non-pathogenic in the *in vivo* model of infection, including the IFN-γ KO mice known to be highly susceptible to *T*. *cruzi* infection. However, in contrast to the results observed during *in vitro* infection, restoration of IPCS activity did not restore the parasite pathogenicity or its ability to survive in the bloodstream of infected animals. Initially, we considered the possibility that the addback trypomastigotes may not be expressing appropriated levels of TcIPCS enzyme, since the pROCK expression vector was designed for expression in epimastigotes. However, the results of western blot analyses showed this was not the case. In addition, metabolic labelling and TLC analyses showed that the addback cell line produced similar amounts of IPC when compared to WT epimastigotes. It has been described that prolonged *in vitro* cultivation of the CL Brener strain results in the selection of parasites with low infection capacity in immunocompetent animals [56]. We tested the possibility that the number of bloodstream trypomastigotes in mice infected with the addback cell line was below the limit of detection of the method, which is approximately 10^4^ parasites/mL using the more sensitive PCR assay. Indeed, the presence of significant higher level of addback parasites in the hearth tissue of infected mice compared to the TcIPCS KO indicated that re-expression of IPCS partially rescued the *in vivo* infection capacity. The partial phenotype rescue in the in vivo model of infection also suggested that the complete reversion of the attenuated phenotype may require factors other than the restoration of IPC production. It is noteworthy that, in addition of the lack of IPC, phosphatidylethanolamine and phosphatidylserines were significantly altered as a consequence of TcIPCS deletion. Finally, although unlikely, the possibility of off-target genomic alterations due to the Cas9 activity cannot be excluded. However, the possibility of off-target effects was significantly reduced because we used the strategy of Cas9 transfection as a ribonucleoprotein (RNP) complex. In totally, the results from both *in vitro* and *in vivo* infection assays with the *TcIPCS* KO mutants strengthen the drive for further investigation of TcIPCS and other enzymes involved in *T*. *cruzi* SL biosynthesis, not only to better understand the role of IPC in *T*. *cruzi* virulence but, also, and more importantly, to consider them as potential targets for the development of new drugs for Chagas disease.

In line with this, using the *TcIPCS* null mutants, we investigated the drugability of sphingolipid metabolism by testing two compounds that have been successfully tested as potential new antileishmanial chemotherapy (benzazepanes [21] and tamoxifen [48]). However, neither compound was indicated as having a target direct effect in *T*. *cruzi*. A third compound, clemastine fumarate [38], recently described as an inhibitor of *Leishmania* IPCS remains to be tested using our mutants. These apparent differences in drug susceptibility came as no surprise when considered the evolutionary divergence between the *T*. *cruzi* and *Leishmania* enzymes, as indicated by our sequence alignment analyses. Similar findings have been described for the antifungal aureobasidin A, which inhibits the *Saccharomyces cerevisiae* IPCS (AUR1p), but is inactive against protozoan IPCS from *L*. *major* and *Toxoplasma gondii* [57]. Further indicating divergence, unlike the *L*. *major* gene, the *T*. *cruzi IPCS* gene does not complement the loss of AUR1p in yeast [22]. In conclusion, although the TcIPCS can still be considered a viable target for the development of a much-needed improved chemotherapy to treat Chagas disease, further studies, including additional statistical analyses, are required to define the role of the enzyme and its product, IPC, for parasite differentiation and infection capacity.

## Supporting information

S1 Fig A) Amino acid sequence alignment of *TcIPCS* (TcCLB.506885.124) and the homologous proteins in *T*. *brucei* (*TbSLS1*: Tb927.9.9410) and Leishmania (*LmF*.*IPCS*: LmjF.35.4990). Transmembrane domains and the second luminal loop are colored in grey and pink, respectively. Catalytic triad and the residue determinant of substrate selectivity are highlighted in blue. B) Alignment *of TcIPCS*, *TbSLS1* and *LmF*.*IPCS* predicted models.
(TIF)

S2 Fig A) Schematic representation of the TOPO-5’_UTR_IPCS-HX1-Neo-GAPDH-3’_UTR_IPCS, the vector used as PCR template for the HDR DNA donor that is composed by a neomycin resistance cassette flanked by upstream and downstream *TcIPCS* regions. The set of primers used for HDR DNA donor construction are represented in the vector diagram. B) PCR showing genotyping of WT and two selected clones of *TcIPCS* knockout epimastigote (KO4 and KO8), demonstrating disruption of the *IPCS* gene and insertion of the donor sequence. C) Negative ion ES-MS lipidomic analysis of WT and *TcICPS* KO4 lipid extracts. Spectra show survey scans (750-950m/z) of WT and KO4 epimastigotes showing a heterogeneous mixture of PI, IPC, PS and PE species, with low abundance lipids better identified compared to the profile shown in Fig 2D.
(TIF)

S3 Fig Sensitivity of epimastigotes to other benzazepanes. WT and *TcIPCS*-KO epimastigotes were grown in presence of increasing concentrations of (A) compound 2 or (B) compound 7a. Viability of the cells was determined by Alamar blue assay. Data points are mean values ± SD of three determinations. Representative experiments performed in triplicates are shown. IC_50_ values are the mean ± SD of at least 3 independent replicates. There were no statistically significant differences between IC_50_ values, as analyzed by Krustal-Wallis.
(TIF)

S4 Fig (A-C) Fluorescence microscopy analyses showing the immunostaining using anti-HA antibody (green), indicate TclPCS protein localization only in the addback parasites (C). (D) Control using addback parasites stain only anti-IgG antibody conjugated with Alexa Fluor 488 (2nd AB) and DAPI, shows absence of autofluorescence of secondary antibody.
(TIF)

S5 Fig Fluorescence microscopy analyses of WT (A), *TcIPCS-*KO8 (B) and AB1 (C) *T*. *cruzi* epimastigotes incubated with NBD C_6_-Ceramide to stain ceramide rich regions (red), such as the Golgi apparatus.
(TIF)

S6 Fig Western blot analysis of HA-tagged IPCS from culture-derived trypomastigotes from WT and *IPCS*-addback cell line (AB1). Arrow indicates the HA-tagged IPCS protein. (TIF)

S1 Table Primers used in this study. Position of each primer is indicated by the letters F (Forward) and R (Reverse). Underlined bases indicate restriction sites for enzyme cleavage.
(PDF)

S2 Table Raw data. (XLSX)
