# Supplementary figures and images for: Disruption of the inositol phosphorylceramide synthase gene affects Trypanosoma cruzi differentiation and infection capacity
Source: PLoS Negl Trop Dis. 2023 Sep 20;17(9):e0011646. doi: 10.1371/journal.pntd.0011646 (PMC10545103; doi:10.1371/journal.pntd.0011646)

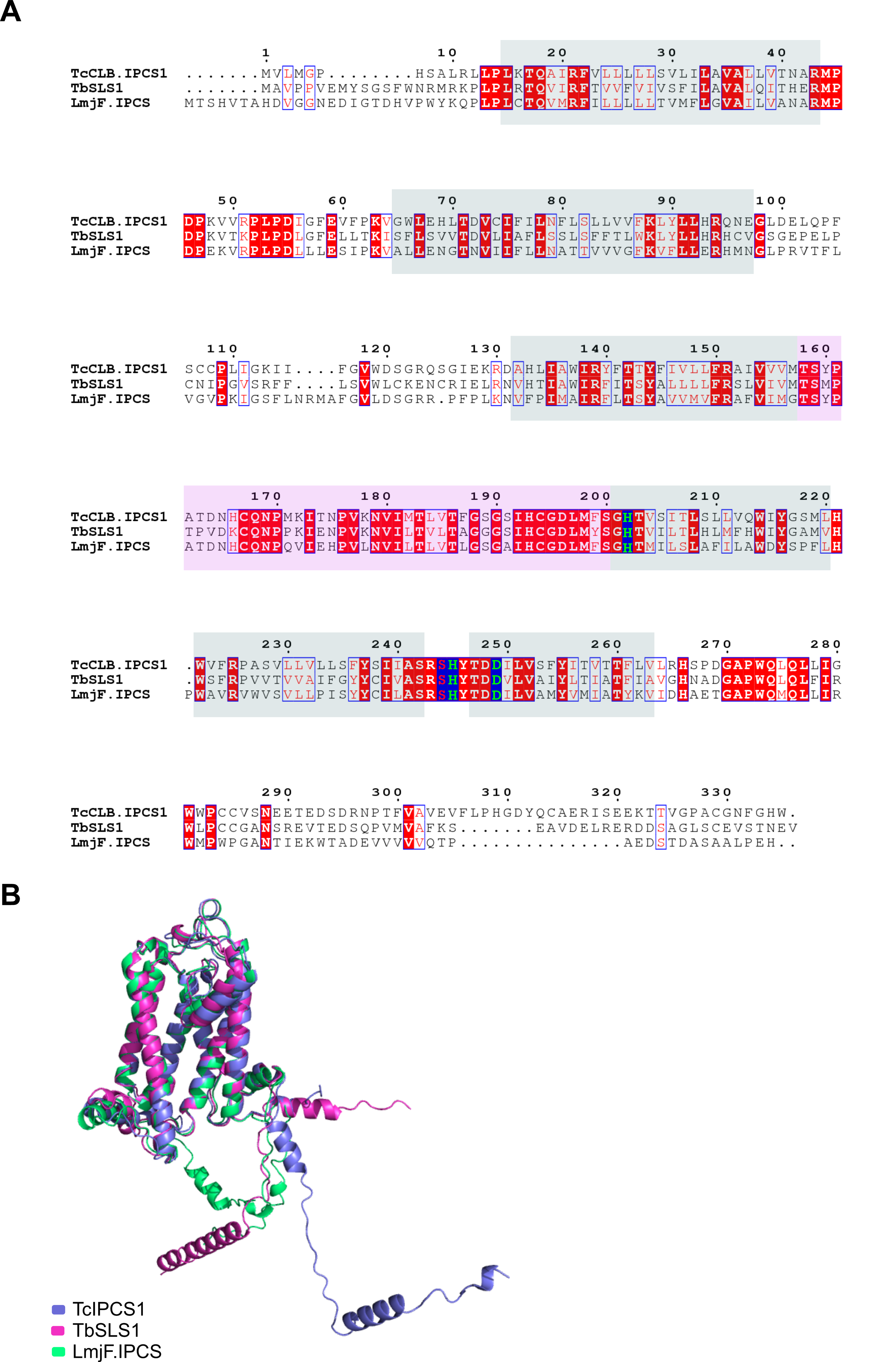

Supplement: S1 Fig — A) Amino acid sequence alignment of TcIPCS (TcCLB.506885.124) and the homologous proteins in T. brucei (TbSLS1: Tb927.9.9410) and Leishmania (LmF.IPCS: LmjF.35.4990). Transmembrane domains and the second luminal loop are colored in grey and pink, respectively. Catalytic triad and the residue determinant of substrate selectivity are highlighted in blue. B) Alignment of TcIPCS, TbSLS1 and LmF.IPCS predicted models. (TIF) [file pntd.0011646.s001.tif]

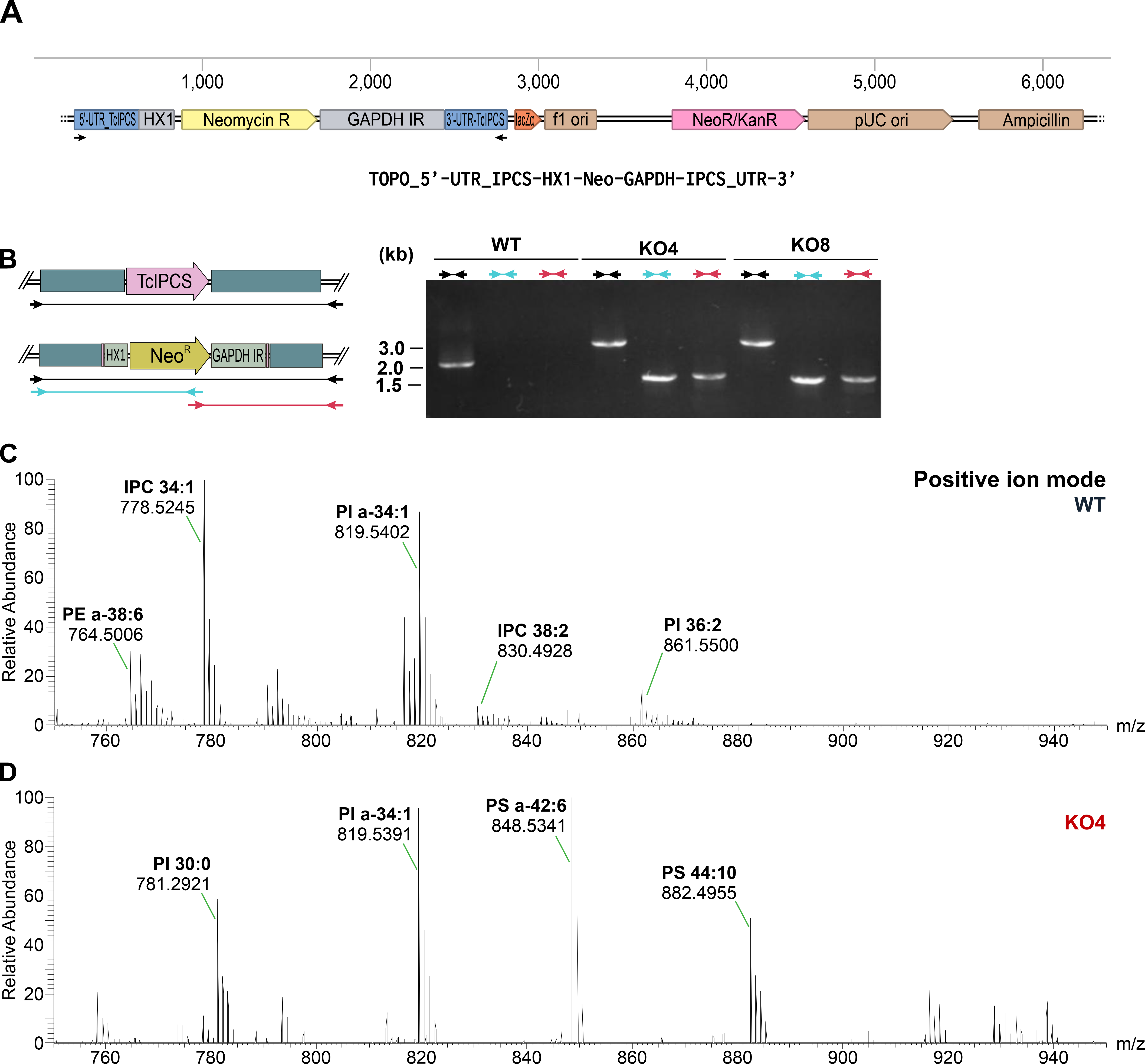

Supplement: S2 Fig — A) Schematic representation of the TOPO-5’_UTR_IPCS-HX1-Neo-GAPDH-3’_UTR_IPCS, the vector used as PCR template for the HDR DNA donor that is composed by a neomycin resistance cassette flanked by upstream and downstream TcIPCS regions. The set of primers used for HDR DNA donor construction are represented in the vector diagram. B) PCR showing genotyping of WT and two selected clones of TcIPCS knockout epimastigote (KO4 and KO8), demonstrating disruption of the IPCS gene and insertion of the donor sequence. C) Negative ion ES-MS lipidomic analysis of WT and TcICPS KO4 lipid extracts. Spectra show survey scans (750-950m/z) of WT and KO4 epimastigotes showing a heterogeneous mixture of PI, IPC, PS and PE species, with low abundance lipids better identified compared to the profile shown in Fig 2D. (TIF) [file pntd.0011646.s002.tif]

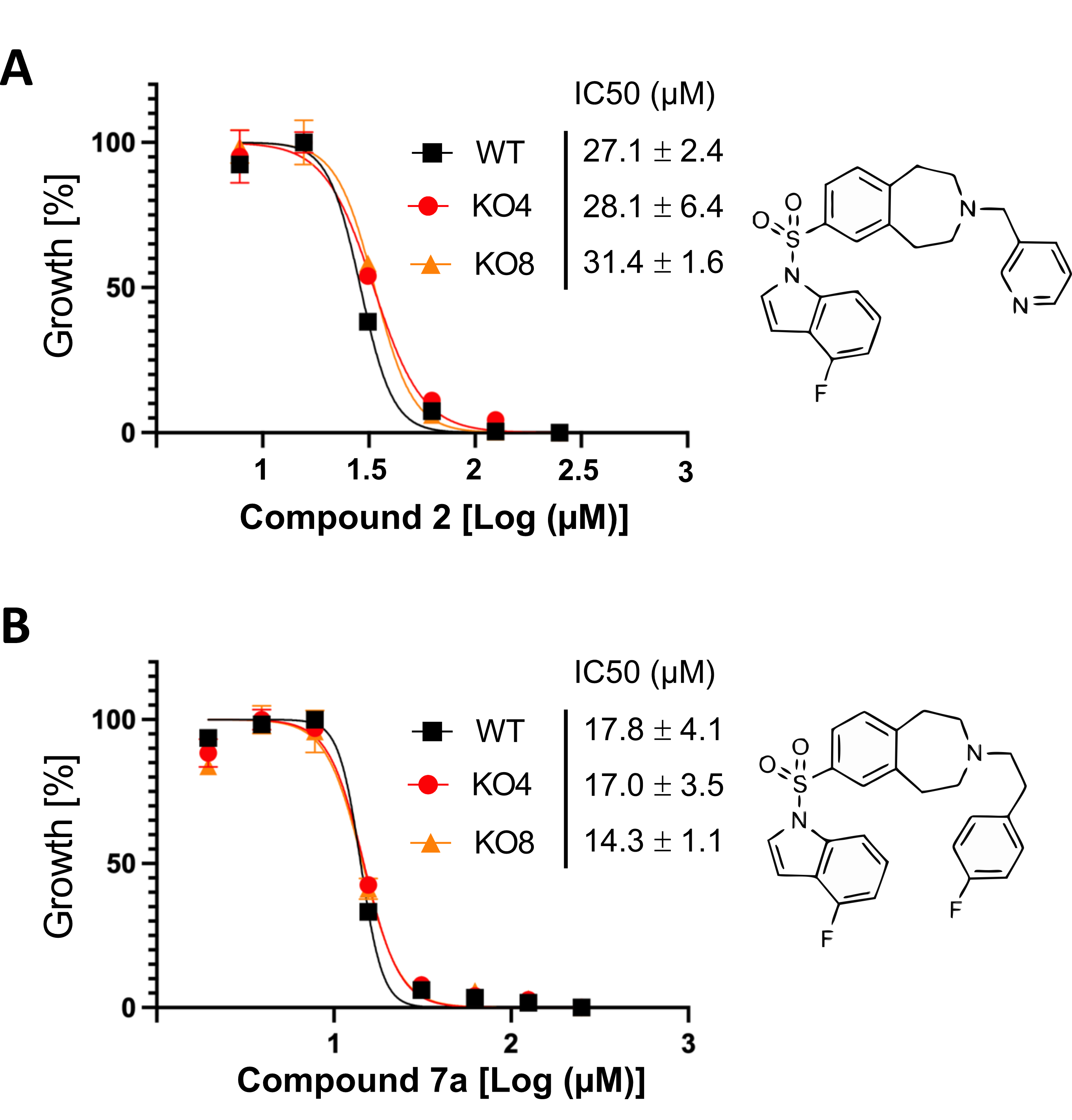

Supplement: S3 Fig — Sensitivity of epimastigotes to other benzazepanes. WT and TcIPCS-KO epimastigotes were grown in presence of increasing concentrations of (A) compound 2 or (B) compound 7a. Viability of the cells was determined by Alamar blue assay. Data points are mean values ± SD of three determinations. Representative experiments performed in triplicates are shown. IC50 values are the mean ± SD of at least 3 independent replicates. There were no statistically significant differences between IC50 values, as analyzed by Krustal-Wallis. (TIF) [file pntd.0011646.s003.tif]

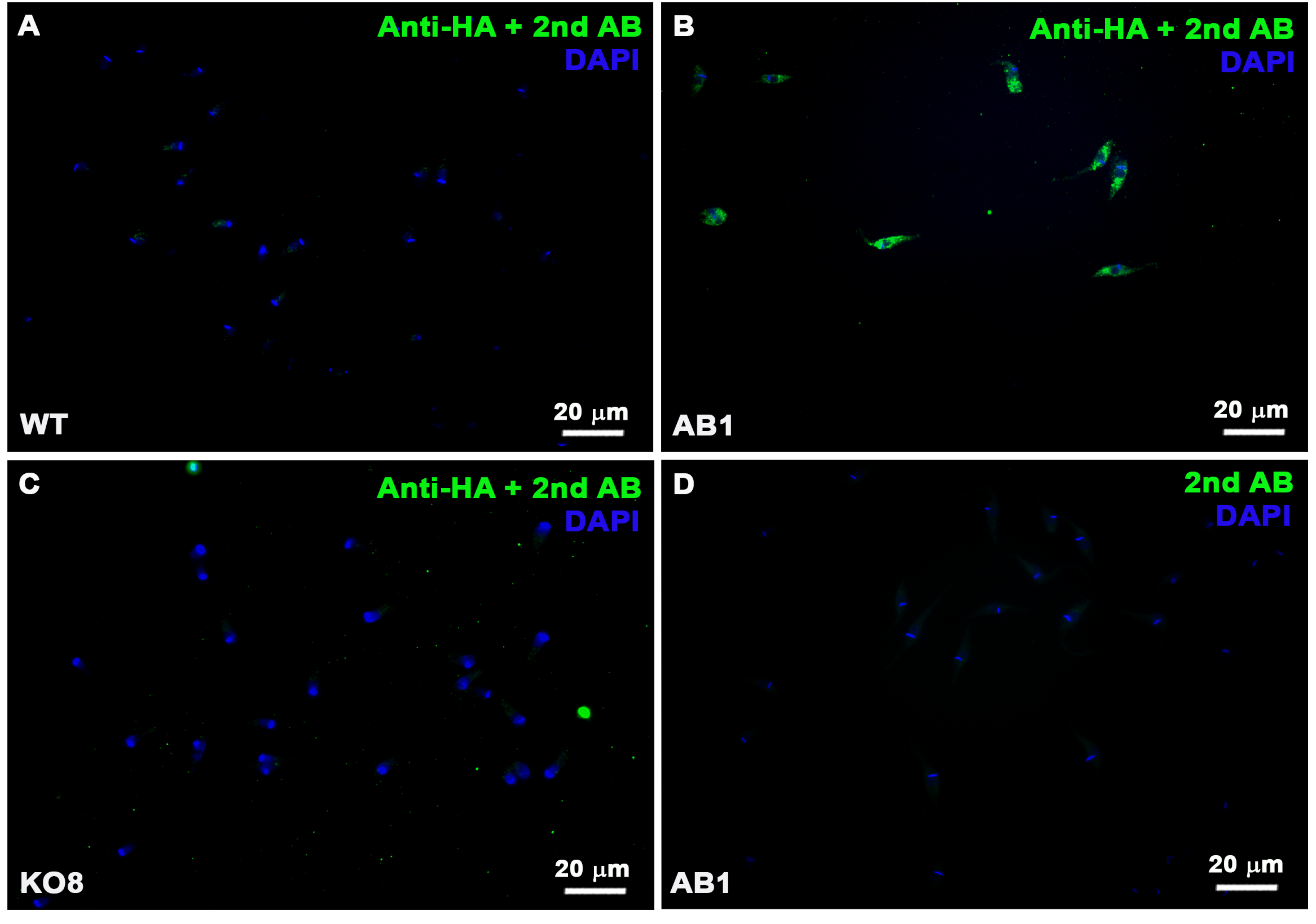

Supplement: S4 Fig — (A-C) Fluorescence microscopy analyses showing the immunostaining using anti-HA antibody (green), indicate TclPCS protein localization only in the addback parasites (C). (D) Control using addback parasites stain only anti-IgG antibody conjugated with Alexa Fluor 488 (2nd AB) and DAPI, shows absence of autofluorescence of secondary antibody. (TIF) [file pntd.0011646.s004.tif]

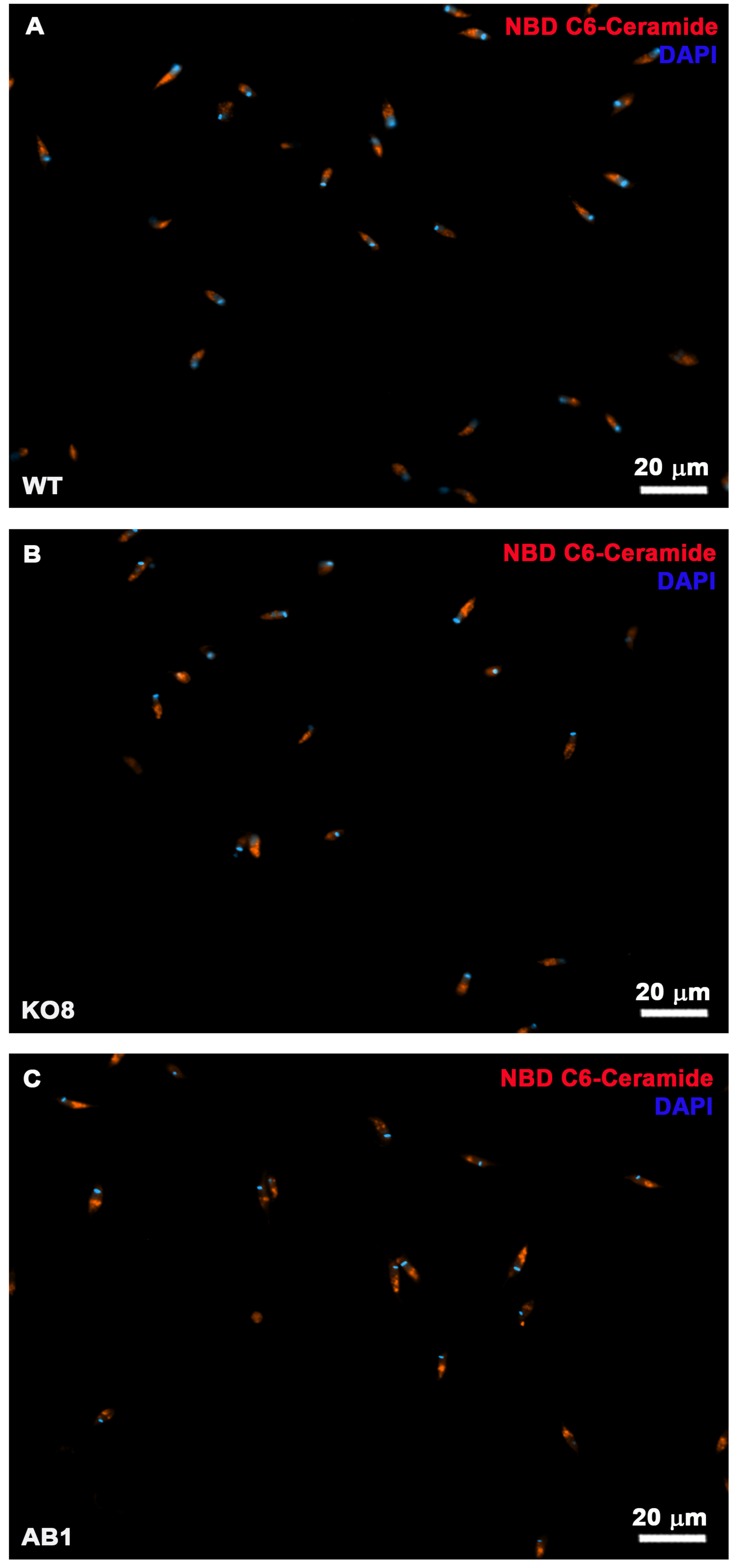

Supplement: S5 Fig — Fluorescence microscopy analyses of WT (A), TcIPCS-KO8 (B) and AB1 (C) T. cruzi epimastigotes incubated with NBD C6-Ceramide to stain ceramide rich regions (red), such as the Golgi apparatus. (TIF) [file pntd.0011646.s005.tif]

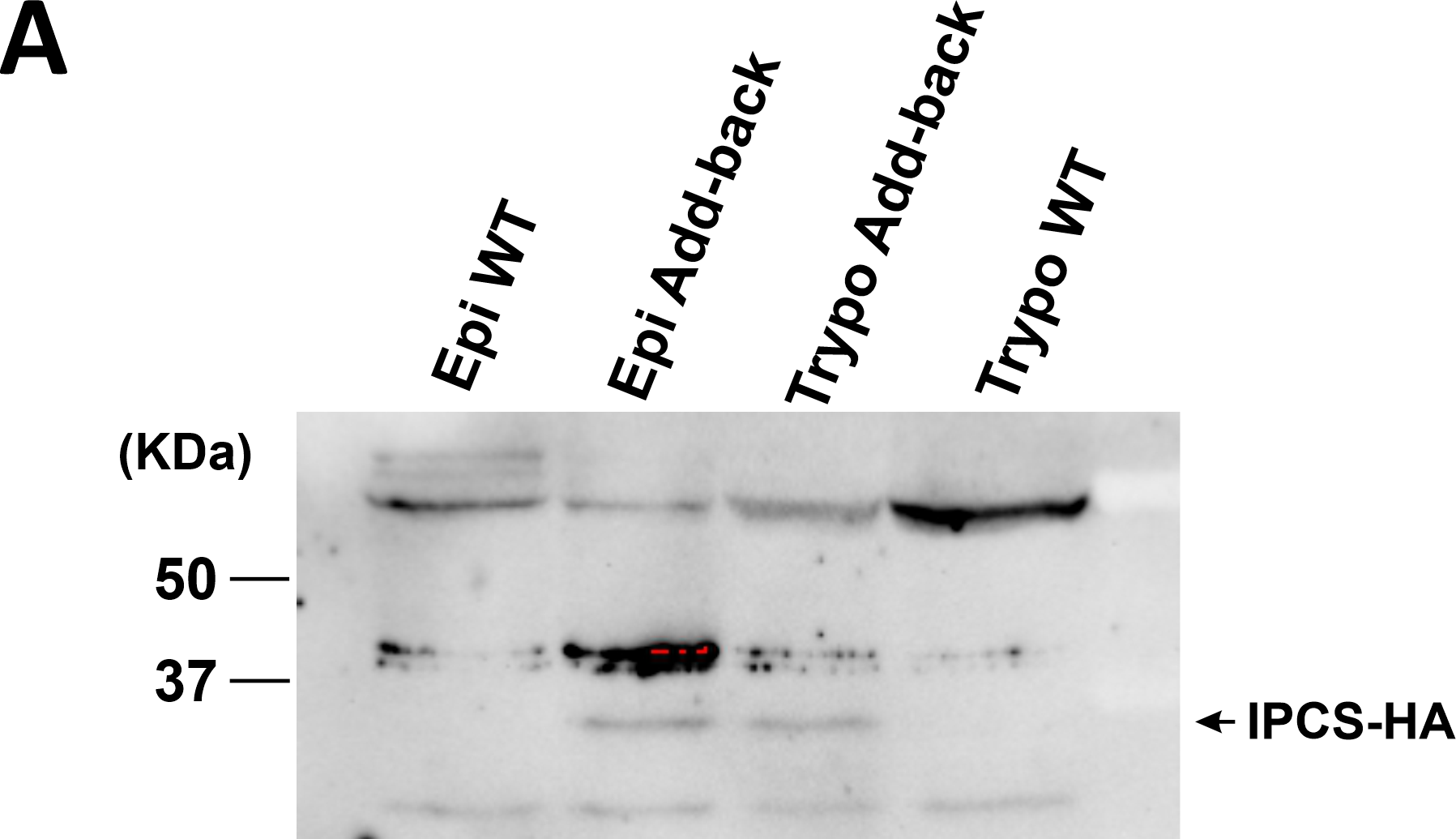

Supplement: S6 Fig — (TIF) [file pntd.0011646.s006.tif]
