# Supplementary material for: Disruption of the inositol phosphorylceramide synthase gene affects Trypanosoma cruzi differentiation and infection capacity
Source: PLoS Negl Trop Dis. 2023 Sep 20;17(9):e0011646. doi: 10.1371/journal.pntd.0011646 (PMC10545103; doi:10.1371/journal.pntd.0011646)
Supplement: S1 Table — Position of each primer is indicated by the letters F (Forward) and R (Reverse). Underlined bases indicate restriction sites for enzyme cleavage. (PDF) [file pntd.0011646.s007.pdf]

S1 table. Primers used in this study. Position of each primer is indicated by the letter F (Forward) and R (Reverse). Underlined bases indicate restriction sites for cleavage.

Purpose	Primer number and name	Sequence (5'→ 3')
**sgRNA amplification**	**1:** TcIPCS-sgRNA-5'-1 F	GGAGGCCGGAGAATTGTAATACG<br>ACTCACTATAGGAAGCAAAAACA<br>GCAGCAAAAAGTTTTAGTACTCT<br>GGAAACAGAATC
	**2:** TcIPCs-sgRNA-3' F	GGAGGCCGGAGAATTGTAATACG<br>ACTCACTATAGGACGACGGTTGG<br>ACCAGCCTGTGTTTTAGTACTCTG<br>GAAACAGAATC
	**3:** Sa-sgRNA scaffold R	TTGAACAACCGCTCTAAAAAAA
**HDR Donor DNA**	**4:** TcIPCS-HDR-*KpnI* -5' F	TTTGGTACCTACCCTTCTTGTCCG TCCTG
	**5:** TcIPCS-HDR-*SpeI* -5' R	GGGACTAGTATAGCCTGCGTTTTCAGTGG
	**6:** TcIPCS-HDR-*EcoRV*-3' F	TTTGATATCGTGGGAATTTTGGACACTGG
	**7:** TcIPCS-HDR-*XhoI* -3' R	TATCTCGAGCTGCGACTCCCTTCACAAATC
	**8:** TcIPCS-HDR-5' F	TACCCTTCTTGTCCGTCCTG
	**9:** TcIPCS-HDR-3' R	TGCGACTCCCTTCACAAATC
	**10:** TcIPCS-Ext-5' F	TTCTTGACTTGTGCGGGTGA

**TcIPCS KO genotyping**	**11:** TcIPCS-Int-3' F	TCGTATCCTGCAACGGACAA
	**12:** TcIPCS-Ext-3' R	TGCGTCACCTACGACTTACTTCC
	**13:** Neo (+804) R	TCAGAAGAACTCGTCAAGAAGGCG
**pROCK-Hygro-IPCS-HA add back**	**14:** TcIPCS1-XbaI-5' F	<u>TCTAGA</u>ATGGTTTTAATGGGGCCT CATTCTG
	**15:** TcIPCS1::HA-XhoI-3' R	<u>CTCGAG</u>TCAAGCGTAATCTGGTA CGTCGTATGGGTA
**qPCR**	**16:** TcIPCS-mRNA F	CGGTCTCCATCACGCTCAGT
	**17:** TcIPCS-mRNA R	TCGTCCGTGTAGTGGGAACG
	**18:** TcL9-mRNA F	CCTTCACTGCCGTTCGTTGGTTTG
	**19:** TcL9-mRNA R	ATGCGAGAGTGCCGTGTTGATGGT
	**20:** GAPDH F	GTGCGGCTGCTGTCAACAT
	**21:** GAPDH R	AAAGACATGCCCGTCAGCTT
	**22:** Satellite sequence-primer 1	ASTCGG CTG ATC GTT TTC GA (S = G or C)
	**23:** Satellite sequence-primer 2	AATTCCTCC AAGCAGCGG ATA
	**24:** TNF F	CCCTCACACTCAGATCATCTTCT
	**25:** TNF R	GCTACGACGTGGGCTACAG
